# Supplementary material for: Economic evaluations of interventional opportunities for the management of mental–physical multimorbidity: a systematic review
Source: BMJ Open. 2023 Feb 28;13(2):e069270. doi: 10.1136/bmjopen-2022-069270 (PMC9980364; doi:10.1136/bmjopen-2022-069270)
Supplement: Supplementary data [file bmjopen-2022-069270supp001.pdf]

SUPPLEMENTARY FILES

Supplementary File 1: Reporting checklist for systematic review (without a meta-analysis) based on the PRISMA guidelines

		Reporting Item	Page Number
**Title**			
Title	#1	Identify the report as a systematic review	1
**Abstract**			
Abstract	#2	Report an abstract addressing each item in the PRISMA 2020 for Abstracts checklist	2
**Introduction**			
Background/rationale	#3	Describe the rationale for the review in the context of existing knowledge	3
Objectives	#4	Provide an explicit statement of the objective(s) or question(s) the review addresses	3
**Methods**			
Eligibility criteria	#5	Specify the inclusion and exclusion criteria for the review and how studies were grouped for the syntheses	3-4
Information sources	#6	Specify all databases, registers, websites, organisations, reference lists, and other sources searched or consulted to identify studies. Specify the date when each source was last searched or consulted	4
Search strategy	#7	Present the full search strategies for all databases, registers, and websites, including any filters and limits used	4
Selection process	#8	Specify the methods used to decide whether a study met the inclusion criteria of the review, including how many reviewers screened each record and each report retrieved, whether they worked independently, and, if applicable, details of automation tools used in the process	4
Data collection process	#9	Specify the methods used to collect data from reports, including how many reviewers collected data from each report, whether they worked independently, any processes for obtaining or confirming data from study investigators, and, if applicable, details of automation tools used in the process	4
Data items	#10a	List and define all outcomes for which data were sought. Specify whether all results that were compatible with each outcome domain in each study were sought (for example, for all measures, time points, analyses), and, if not, the methods used to decide which results to collect	4
Study risk of bias assessment	#11	Specify the methods used to assess risk of bias in the included studies, including details of the tool(s) used, how many reviewers assessed each study and whether they worked independently, and, if applicable, details of automation tools used in the process	4-5
Effect measures	#12	Specify for each outcome the effect measure(s) (such as risk ratio, mean difference) used in the synthesis or presentation of results	n/a
Synthesis methods	#13a	Describe the processes used to decide which studies were eligible for each synthesis (such as tabulating the study intervention characteristics and comparing against the planned groups for each synthesis (item #5))	5
Synthesis methods	#13b	Describe any methods required to prepare the data for presentation or synthesis, such as handling of missing summary statistics or data conversions	5
Synthesis methods	#13c	Describe any methods used to tabulate or visually display results of individual studies and syntheses	5
Synthesis methods	#13d	Describe any methods used to synthesise results and provide a rationale for the choice(s). If meta-analysis was performed, describe the model(s), method(s) to identify the presence and extent of statistical heterogeneity, and software package(s) used	5
Synthesis methods	#13e	Describe any methods used to explore possible causes of heterogeneity among study results (such as subgroup analysis, meta-regression)	5
Synthesis methods	#13f	Describe any sensitivity analyses conducted to assess robustness of the synthesised results	n/a
Reporting bias assessment	#14	Describe any methods used to assess risk of bias due to missing results in a synthesis (arising from reporting biases)	4-5
Certainty assessment	#15	Describe any methods used to assess certainty (or confidence) in the body of evidence for an outcome	5

Data items	#10b	List and define all other variables for which data were sought (such as participant and intervention characteristics, funding sources). Describe any assumptions made about any missing or unclear information	4
**Results**			
Study selection	#16a	Describe the results of the search and selection process, from the number of records identified in the search to the number of studies included in the review, ideally using a flow diagram (http://www.prisma-statement.org/PRISMAStatement/FlowDiagram)	5
Study selection	#16b	Cite studies that might appear to meet the inclusion criteria, but which were excluded, and explain why they were excluded	5
Study characteristics	#17	Cite each included study and present its characteristics	5
Risk of bias in studies	#18	Present assessments of risk of bias for each included study	5-6
Results of individual studies	#19	For all outcomes, present for each study (a) summary statistics for each group (where appropriate) and (b) an effect estimate and its precision (such as confidence/credible interval), ideally using structured tables or plots	6-8
Results of syntheses	#20a	For each synthesis, briefly summarise the characteristics and risk of bias among contributing studies	6-8
Results of syntheses	#20b	Present results of all statistical syntheses conducted. If meta-analysis was done, present for each the summary estimate and its precision (such as confidence/credible interval) and measures of statistical heterogeneity. If comparing groups, describe the direction of the effect	6-8
Results of syntheses	#20c	Present results of all investigations of possible causes of heterogeneity among study results	6-8
Results of syntheses	#20d	Present results of all sensitivity analyses conducted to assess the robustness of the synthesised results	n/a
Risk of reporting biases in syntheses	#21	Present assessments of risk of bias due to missing results (arising from reporting biases) for each synthesis assessed	6-8
Certainty of evidence	#22	Present assessments of certainty (or confidence) in the body of evidence for each outcome assessed	6-8
**Discussion**			
Results in context	#23a	Provide a general interpretation of the results in the context of other evidence	8-9
Limitations of included studies	#23b	Discuss any limitations of the evidence included in the review	9
Limitations of the review methods	#23c	Discuss any limitations of the review processes used	9
Implications	#23d	Discuss implications of the results for practice, policy, and future research	9-10
**Other information**			
Registration and protocol	#24a	Provide registration information for the review, including register name and registration number, or state that the review was not registered	3
Registration and protocol	#24b	Indicate where the review protocol can be accessed, or state that a protocol was not prepared	3
Registration and protocol	#24c	Describe and explain any amendments to information provided at registration or in the protocol	n/a
Support	#25	Describe sources of financial or non-financial support for the review, and the role of the funders or sponsors in the review	10
Competing interests	#26	Declare any competing interests of review authors	10
Availability of data, code, and other materials	#27	Report which of the following are publicly available and where they can be found: template data collection forms; data extracted from included studies; data used for all analyses; analytic code; any other materials used in the review	10

The PRISMA checklist is distributed under the terms of the Creative Commons Attribution License CC-BY. This checklist was completed on 07 October 2022 using https://www.goodreports.org/, a tool made by the EQUATOR Network in collaboration with Penelope.ai

Supplementary File 2: Search strategy

1. **MEDLINE (Ovid SP)**

No.	Search terms	Results
1	comorbidity/ or multimorbidity/	122184
2	Chronic Disease/	273360
3	(comorbid* or co-morbid*).ab,kf,ti.	184267
4	(multimorbid* or multi-morbid*).ab,kf,ti.	6384
5	(multidisease? or multi-disease? or multi-condition? multicondition? or ((multi or multiple) adj2 (morbid* or ill* or disease? or condition? or syndrom* or diagnos? or disorder?))).ab,kf,ti.	38012
6	((cooccur* or co-occur* or coexist* or co-exist* or multipl* or concord* or discord*) adj3 (disease? or ill* or care or condition? or disorder* or health* or medication* or symptom* or syndrom* or morbid*)).ab,kf,ti.	87329
7	((polypatholog* or poly-patholog* or polymorbid* or poly-morbid* or multipatholog* or multi-patholog* or pluripatholog* or pluri-patholog* or concurrent) adj2 (disease* or illness* or condition* or diagnos#s or morbid*)).ab,kf,ti.	4534
8	(chronic* adj (disease? or ill* or care or condition? or disorder* or health* or medication* or syndrom* or symptom*)).ab,kf,ti.	114969
9	Polypharmacy/	5973
10	(polypharmac* or poly-pharmac* or polymedicat* or poly-medicat*).ab,kf,ti.	9155
11	or/1-10	688494
12	"depress*".ab,ti.	446771
13	Depression/	137109
14	Depressive symptoms.mp.	48782
15	depressive disorder.ab,ti.	27105
16	Depressive Disorder/	74516
17	Depressive Disorder, Major/	34500
18	Major depression.mp.	22963
19	Major depression disorder.mp.	394
20	MDD.ti.	224
21	Sadness/	259
22	melancholia.mp.	1368
23	Emotions/	75999
24	Mental Disorders/	171825
25	"dysthymi*".ab,ti.	2955
26	Dysthymic Disorder/	1163
27	Persistent Depressive Disorder.mp.	96
28	"mood disorder*".ab,ti.	16249
29	Mood Disorders/	15316
30	or/12-29	714111
31	Economics/	27415
32	exp "costs and cost analysis"/	253608
33	Economics, Dental/	1920
34	exp economics, hospital/	25478
35	Economics, Medical/	9182
36	Economics, Nursing/	4012
37	Economics, Pharmaceutical/	3054
38	(economic* or cost or costs or costly or costing or price or prices or pricing or pharmacoeconomic*).ab,ti.	742224
39	(expenditure* not energy).ab,ti.	28993
40	value for money.ab,ti.	1638
41	budget*.ab,ti.	26338
42	or/31-41	894717
43	((energy or oxygen) adj cost).ab,ti.	3663
44	(metabolic adj cost).ab,ti.	1360
45	((energy or oxygen) adj expenditure).ab,ti.	24402
46	or/43-45	28486
47	42 not 46	888486
48	letter.pt.	1118419
49	editorial.pt.	521968

50	historical article.pt.	367453
51	review.pt.	2672874
52	meta analysis.pt.	151585
53	news.pt.	184561
54	comment.pt.	887910
55	cochrane database of systematic reviews.jn.	15444
56	comment on.cm.	887869
57	(systematic review or literature review).ti.	170547
58	or/48-57	5127987
59	47 not 58	704031
60	exp animals/ not humans/	4950657
61	59 not 60	643170
62	11 and 30 and 61	4171

2. CINAHL Plus (EBSCOhost)

No.	Search terms	Results
S1	MH "Comorbidity"	67846
S2	MH "Chronic Disease"	69431
S3	TI (comorbid* or co-morbid* or multimorbid* or multi-morbid*) OR AB (comorbid* or co-morbid* or multimorbid* or multi-morbid*)	82429
S4	(multidisease? or multi-disease? or multi-condition? multicondition? or ((multi or multiple) N2 (morbid* or ill* or disease? or condition? or syndrom* or diagnos? or disorder*)))	18069
S5	((cooccur* or co-occur* or coexist* or co-exist* or multipl* or concord* or discord*) N3 (disease? or ill* or care or condition? or disorder* or health* or medication* or symptom* or syndrom* or morbid*))	41577
S6	((polypatholog* or poly-patholog* or polymorbid* or poly-morbid* or multipatholog* or multi-patholog* or pluripatholog* or pluri-patholog* or concurrent) N2 (disease* or illness* or condition* or diagnos#s or morbid*))	1452
S7	TI (chronic* N0 (disease? or ill* or care or condition? or disorder* or health* or medication* or syndrom* or symptom*)) OR AB (chronic* N0 (disease? or ill* or care or condition? or disorder* or health* or medication* or syndrom* or symptom*))	62168
S8	(polypharmac* or poly-pharmac* or polymedicat* or poly-medicat*)	7308
S9	S1 OR S2 OR S3 OR S4 OR S5 OR S6 OR S7 OR S8	266469
S10	MH "Depression+"	126224
S11	MH "Emotions+"	159155
S12	MM "Mental Disorders"	45553
S13	TI (depress* or dysthymi* or “mood disorder*” or “affective disorder*”) OR AB (depress* or dysthymi* or “mood disorder*” or “affective disorder*”)	173728
S14	S10 OR S11 OR S12 OR S13	360281
S15	MH "Economics+"	899886
S16	MH "Financial Management+"	72445
S17	MH "Financial Support+"	551447
S18	MH "Financing, Organized+"	166964
S19	MH "Business+"	176961
S20	S16 OR S17 or S18 OR S19	898765
S21	S15 NOT S20	115916
S22	MH "Health Resource Allocation"	10024
S23	MH "Health Resource Utilization"	21011
S24	S22 OR S23	30481
S25	S21 OR S24	137018
S26	TI (cost or costs or economic* or pharmacoeconomic* or price* or pricing*) OR AB (cost or costs or economic* or pharmacoeconomic* or price* or pricing*)	263212
S27	S25 OR S26	343360
S28	PT editorial	329356
S29	PT letter	379399
S30	PT commentary	387092
S31	S28 OR S29 OR S30	844698
S32	S27 NOT S31	319691
S33	MH "Animal Studies"	145184
S34	(ZT "doctoral dissertation") or (ZT "masters thesis")	26307

S35	S32 NOT (S33 OR S34)	315837
S36	S9 AND S14 AND S35	2577

3. PsycINFO (EBSCOhost)

No.	Search terms	Results
S1	DE "Comorbidity"	55731
S2	DE "Chronic Illness"	12829
S3	TI (comorbid* or co-morbid* or multimorbid* or multi-morbid*) OR AB (comorbid* or co-morbid* or multimorbid* or multi-morbid*)	63695
S4	(multidisease? or multi-disease? or multi-condition? multicondition? or ((multi or multiple) N2 (morbid* or ill* or disease? or condition? or syndrom* or diagnos? or disorder*)))	12286
S5	((cooccur* or co-occur* or coexist* or co-exist* or multipl* or concord* or discord*) N3 (disease? or ill* or care or condition? or disorder* or health* or medication* or symptom* or syndrom* or morbid*))	34010
S6	((polypatholog* or poly-patholog* or polymorbid* or poly-morbid* or multipatholog* or multi-patholog* or pluripatholog* or pluri-patholog* or concurrent) N2 (disease* or illness* or condition* or diagnos#s or morbid*))	1117
S7	TI (chronic* N0 (disease? or ill* or care or condition? or disorder* or health* or medication* or syndrom* or symptom*)) OR AB (chronic* N0 (disease? or ill* or care or condition? or disorder* or health* or medication* or syndrom* or symptom*))	33537
S8	(polypharmac* or poly-pharmac* or polymedicat* or poly-medicat*)	3094
S9	S1 OR S2 OR S3 OR S4 OR S5 OR S6 OR S7 OR S8	153121
S10	DE "Major Depression" OR DE "Anaclitic Depression" OR DE "Dysthymic Disorder" OR DE "Endogenous Depression" OR DE "Late Life Depression" OR DE "Postpartum Depression" OR DE "Reactive Depression" OR DE "Recurrent Depression" OR DE "Treatment Resistant Depression"	145751
S11	DE "Depression (Emotion)"	26441
S12	DE "Sadness"	2491
S13	DE "Mental Disorders"	138411
S14	DE "Affective Disorders"	15106
S15	TI (depress* or dysthymi* or “mood disorder*” or “affective disorder*”) OR AB (depress* or dysthymi* or “mood disorder*” or “affective disorder*”)	344586
S16	S10 OR S11 OR S12 OR S13 OR S14 OR S15	465624
S17	Costs and Cost Analysis	19397
S18	Cost Containment	1221
S19	TI (economic N2 evaluation) OR AB (economic N2 evaluation)	2068
S20	TI (economic N2 analy*) OR AB (economic N2 analy*)	2423
S21	TI (economic N2 (study OR studies)) OR AB (economic N2 (study OR studies))	2378
S22	TI (cost N2 evaluation*) OR AB (cost N2 evaluation*)	717
S23	TI (cost N2 analy*) OR AB (cost N2 analy*)	5034
S24	TI (cost N2 (study or studies)) OR AB (cost N2 (study or studies))	2789
S25	TI (cost N2 effective*) OR AB (cost N2 effective*)	17524
S26	TI (cost N2 benefit*) OR AB (cost N2 benefit*)	9147
S27	TI (cost N2 utili*) OR AB (cost N2 utili*)	2604
S28	TI (cost N2 minimi*) OR AB (cost N2 minimi*)	1040
S29	TI (cost N2 consequence*) OR AB (cost N2 consequence*)	465
S30	TI (cost N2 comparison*) OR AB (cost N2 comparison*)	422
S31	TI (cost N2 identificat*) OR AB (cost N2 identificat*)	91
S32	TI (pharmacoeconomic* or pharmaco-economic*) OR AB (pharmacoeconomic* or pharmaco-economic*)	336
S33	S1 OR S2 OR S3 OR S4 OR S5 OR S6 OR S7 OR S8 OR S9 OR S10 OR S11 OR S12 OR S13 OR S14 OR S15 OR S16	49801
S34	TI (task N2 cost*) OR AB (task N2 cost*)	1044
S35	TI (switch* N2 cost*) OR AB (switch* N2 cost*)	1533
S36	TI (metabolic N2 cost) OR AB (metabolic N2 cost)	232
S37	TI ((energy or oxygen) N0 cost) OR AB ((energy or oxygen) N0 cost)	446
S38	TI ((energy or oxygen) N0 expenditure) OR AB ((energy or oxygen) N0 expenditure)	2837
S39	S18 OR S19 OR S20 OR S21 OR S22	5626

S40	TI (animal or animals or rat or rats mouse or mice or hamster or hamsters or dog or dogs or cat or cats or bovine or sheep or ovine or pig or pigs) OR AB (animal or animals or rat or rats or mouse or mice or hamster or hamsters or dog or dogs or cat or cats or bovine or sheep or ovine or pig or pigs) OR DE (animal or animals or rat or rats mouse or mice or hamster or hamsters or dog or dogs or cat or cats or bovine or sheep or ovine or pig or pigs)	429599
S41	PZ editorial	44303
S42	PZ letter	24815
S43	PT dissertation abstract	528699
S44	S24 OR S25 OR S26 OR S27	1004717
S45	IS (0003-4819 or 0003-9926 or 0959-8146 or 0098-7484 or 0140-6736 or 0028-4793 or 1469-493X)	13605
S46	S17 NOT (S23 OR S28 OR S29)	41917
S47	S9 AND S16 AND S46	695

4. Cochrane Central Register of Controlled Trials (CENTRAL) (Wiley)

No.	Search terms	Results
#1	[mh ^comorbidity] or [mh ^multimorbidity]	3821
#2	[mh ^"chronic disease"]	13630
#3	(comorbid* or co-morbid*):ti,ab	22372
#4	(multimorbid* or multi-morbid*):ti,ab	609
#5	(multidisease? or multi-disease? or multi-condition? multicondition? or ((multi or multiple) near/2 (morbid* or ill* or disease? or condition? or syndrom* or diagnos? or disorder?))):ti,ab	3685
#6	((cooccur* or co-occur* or coexist* or co-exist* or multipl* or concord* or discord*) near/3 (disease* or ill* or care or condition? or disorder* or health* or medication* or symptom* or syndrom* or morbid*)):ti,ab	8900
#7	((polypatholog* or poly-patholog* or polymorbid* or poly-morbid* or multipatholog* or multi-patholog* or pluripatholog* or pluri-patholog* or concurrent) near/2 (disease* or illness* or condition* or diagnos?s or morbid*)):ti,ab	519
#8	(chronic* next (disease? or ill* or care or condition? or disorder* or health* or medication* or syndrom* or symptom*)):ti,ab	14624
#9	[mh ^polypharmacy]	237
#10	(polypharmac* or poly-pharmac* or polymedicat* or poly-medicat*):ti,ab	1024
#11	{OR #1-#10}	60341
#12	[mh Depression]	13714
#13	[mh "Depressive Disorder"]	13119
#14	[mh Emotions]	28516
#15	[mh ^"Mental disorders"]	4063
#16	[mh "Mood disorders"]	13859
#17	(depress* or dysthymi* or "mood disorder*" or "affective disorder*" or "Persistent Depressive Disorder"):ti,ab,kw	97565
#18	{OR #12-#17}	113806
#19	[mh "Health Care Economics and Organizations"]	23351
#20	[mh Economics]	13515
#21	(economic* or cost or costs or costly or costing or price or prices or pricing or pharmacoeconomic*):ti,ab,kw	90336
#22	(expenditure* not energy)	2401
#23	("value for money"):ti,ab,kw	271
#24	(budget*):ti,ab,kw	1274
#25	health economics	2735
#26	health resource allocation	20
#27	health resource utilization	318
#28	cost consequence*	171
#29	{OR #19-#28}	102424
#30	(energy or oxygen) next (cost or expenditure):ti,ab,kw	5322
#31	(metabolic next cost):ti,ab,kw	136
#32	#30 or #31	5432
#33	#29 NOT #32	101608
#34	[mh "Animal Experimentation"]	2
#35	[mh "Human Experimentation"]	143
#36	#33 NOT (#34 or #35)	101578

#37	#11 AND #18 AND #36 in Trials	1498

5. SCOPUS

No.	Search terms	Results
1	((TITLE-ABS (comorbid* OR co-morbid* OR multimorbid* OR multi-morbid* OR "chronic disease")) OR (TITLE-ABS (multidisease* OR multi-disease* OR multi-condition* OR multicondition*)) OR (TITLE-ABS ((multi OR multiple) W/2 (morbid* OR ill* OR disease* OR condition* OR syndrom* OR diagnos* OR disorder*))) OR (TITLE-ABS ((cooccur* OR co-occur* OR coexist* OR co-exist* OR multipl* OR concord* OR discord*) W/3 (disease* OR ill* OR care OR condition* OR disorder* OR health* OR medication* OR symptom* OR syndrom* OR morbid*))) OR (TITLE-ABS ((polypatholog* OR poly-patholog* OR polymorbid* OR poly-morbid* OR multipatholog* OR multi-patholog* OR pluripatholog* OR pluri-patholog* OR concurrent) W/2 (disease* OR illness* OR condition* OR diagnosis OR morbid*))) OR (TITLE-ABS (chronic* W/1 (disease* OR ill* OR care OR condition* OR disorder* OR health* OR medication* OR syndrom* OR symptom*))) OR (TITLE-ABS (polypharmac* OR poly-pharmac* OR polymedicat* OR poly-medicat*))) AND (TITLE-ABS(depress* OR {MDD} OR sadness OR melancholia OR emotions OR "mental disorder" OR dysthymi* OR "mood disorder*")) AND ((TITLE-ABS("Costs and Cost Analysis")) OR (TITLE-ABS("Cost Containment")) OR (TITLE-ABS(economic W/2 (evaluation OR analy* OR study OR studies))) OR (TITLE-ABS(cost* W/2 (effective* OR utili* OR benefit* OR minimi* OR evaluation* OR analy* OR study OR studies OR consequence* OR comparison* OR efficienc* OR identificat*))) OR (TITLE-ABS (budget* OR economic* OR pharmacoeconomic* OR "pharmaco-economic*")))	3015

6. Web of Science Core Collection

No.	Search terms	Results
1	TI=(comorbid* OR co-morbid* OR multimorbid* OR multi-morbid* OR multidisease* OR multi-disease* OR multi-condition* OR multicondition*)	41154
2	TI=((multi OR multiple) NEAR/2 (morbid* OR ill* OR disease* OR condition* OR syndrom* OR diagnos* OR disorder*))	20523
3	TI=((cooccur* OR co-occur* OR coexist* OR co-exist* OR multipl* OR concord* OR discord*) NEAR/3 (morbid* OR ill* OR disease* OR condition* OR syndrom* OR diagnos* OR disorder*))	25361
4	TI=((polypatholog* OR poly-patholog* OR polymorbid* OR poly-morbid* OR multipatholog* OR multi-patholog* OR pluripatholog* OR pluri-patholog* OR concurrent OR con-current) NEAR/2 (morbid* OR ill* OR disease* OR condition* OR syndrom* OR diagnos* OR disorder*))	1027
5	TI=((chronic*) NEAR/1 (disease* OR ill* OR care OR condition* OR disorder* OR health* OR medication* OR syndrom* OR symptom*))	89527
6	TI=(polypharmac* OR poly-pharmac* OR polymedicat* OR poly-medicat*)	3727
7	#6 OR #5 OR #4 OR #3 OR #2 OR #1	162569
8	TS=(depress* OR "MDD" OR sadness OR melancholia OR emotions OR dysthymi* OR "mental disorder" OR "mood disorder*" OR "affective disorder*")	911185
9	TS=(costs and cost analysis OR cost containment OR budget* OR economic* OR pharmacoeconomic* OR "pharmaco-economic*" OR price OR prices OR pricing OR fee OR fees)	2149872
10	TS=((economic) NEAR/2 (evaluation OR analy* OR study OR studies))	96305
11	TS=((cost*) NEAR/2 (effective* OR utili* OR benefit* OR minimi* OR evaluation* OR analy* OR study OR studies OR consequence* OR comparison* OR efficienc* OR identificat*))	528311
12	#9 OR #10 OR #11	2435312
13	#7 AND #8 AND #12	801

7. NHS Economic Evaluation Database (EED), HTA

Note: Bibliographic records were published on NHS EED until 31st March 2015.

No.	Search terms	Results
1	MeSH descriptor Comorbidity in NHSEED,HTA	166
2	MeSH descriptor Chronic Disease EXPLODE ALL TREES in NHSEED,HTA	469
3	(comorbid* or co-morbid*) in NHSEED, HTA	787

4	(multimorbid* or multi-morbid*) in NHSEED, HTA	6
5	(multidisease* or multi-disease* or multi-condition* multicondition* or ((multi or multiple) NEAR2 (morbid* or ill* or disease* or condition* or syndrom* or diagnos* or disorder*))) in NHSEED, HTA	71
6	((cooccur* or co-occur* or coexist* or co-exist* or multipl* or concord* or discord*) NEAR3 (disease* or ill* or care or condition? or disorder* or health* or medication* or symptom* or syndrom* or morbid*)) in NHSEED, HTA	128
7	((polypatholog* or poly-patholog* or polymorbid* or poly-morbid* or multipatholog* or multi-patholog* or pluripatholog* or pluri-patholog* or concurrent) NEAR2 (disease* or illness* or condition* or diagnos?s or morbid*)) in NHSEED, HTA	27
8	(multifactorial disease* or dual diagnosis) in NHSEED, HTA	4
9	(chronic* NEAR1 (disease* or ill* or care or condition* or disorder* or health* or medication* or syndrom* or symptom*)) in NHSEED, HTA	1112
10	MeSH descriptor Polypharmacy EXPLODE ALL TREES in NHSEED,HTA	8
11	(polypharmac* or poly-pharmac* or polymedicat* or poly-medicat*) in NHSEED, HTA	20
12	#1 OR #2 OR #3 OR #4 OR #5 OR #6 OR #7 OR #8 OR #9 OR #10 OR #11	2007
13	MeSH descriptor Depression EXPLODE ALL TREES in NHSEED,HTA	182
14	MeSH descriptor Depressive Disorder EXPLODE ALL TREES in NHSEED,HTA	337
15	MeSH descriptor Emotions EXPLODE ALL TREES in NHSEED,HTA	100
16	MeSH descriptor Mental Disorders in NHSEED,HTA	215
17	MeSH descriptor Mood Disorders EXPLODE ALL TREES in NHSEED,HTA	352
18	(depress* or dysthymi* or (mood disorder*) or (affective disorder*) or (Persistent Depressive Disorder)) in NHSEED, HTA	939
19	#13 OR #14 OR #15 OR #16 OR #17 OR #18	1152
20	#12 AND #19	163

Supplementary File 3: Characteristics of excluded studies

**Study**	**Reason for exclusion**
Achilla et al. 2013[1]	Conference abstract only
Walker et al. 2013[2]	Conference abstract only
Ladapo et al. 2012[3]	Brief research letter
	Does not meet the inclusion criteria (2 articles)
Pan et al. 2015[4]	There is a lack of clarity in the study regarding the presence of chronic conditions. The authors have stated depressed patients with and without comorbid painful physical symptoms (PPS) that include headaches, back pains, gastrointestinal pains, and musculoskeletal pains. However, it was not clear whether these PPS were chronic conditions.
Panagioti et al. 2018[5]	Although this study had a depression component, it was not the primary focus in multimorbidity. Depression was one of the secondary outcome measures of the study.

References of excluded studies:

1. Achilla E, McCrone P, Phillips R, *et al.* UPBEAT-UK: cost-effectiveness of nurse-led case management and usual care for patients with coronary heart disease and co-morbid depression. *Journal of Mental Health Policy and Economics* 2013;**16**:S1.

2. Walker S, Walker J, Richardson G, *et al.* Cost-Effectiveness of the Systematic Identification and Treatment of Comorbid Major Depression for People with Chronic Diseases: The Example of Cancer. *Value Health* 2013;**16**:A414. doi:10.1016/j.jval.2013.08.523

3. Ladapo JA, Shaffer JA, Fang Y, *et al.* Cost-effectiveness of enhanced depression care after acute coronary syndrome: results from the Coronary Psychosocial Evaluation Studies randomized controlled trial. *Arch Intern Med* 2012;**172**:1682–4. doi:10.1001/archinternmed.2012.4448

4. Pan Y-J, Pan C-H, Chan H-Y, *et al.* Depression and pain: an appraisal of cost effectiveness and cost utility of antidepressants. *J Psychiatr Res* 2015;**63**:123–31. doi:10.1016/j.jpsychires.2015.01.019

5. Panagioti M, Reeves D, Meacock R, *et al.* Is telephone health coaching a useful population health strategy for supporting older people with multimorbidity? An evaluation of reach, effectiveness and cost-effectiveness using a 'trial within a cohort'. *BMC Med* 2018;**16**:80. doi:10.1186/s12916-018-1051-5

Supplementary File 4: Characteristics of included studies

Study	Location & Setting	Population	Disease conditions	Intervention	Comparator	Costs analysed	Outcomes assessed
**Study details:** Aragones, E., Sanchez-Iriso, E., Lopez-Cortacans, G., Tome-Pires, C., Rambla, C. & Sanchez-Rodriguez, E. 2020. Cost-effectiveness of a collaborative care program for managing major depression and chronic musculoskeletal pain in primary care: Economic evaluation alongside a randomized controlled trial. J Psychosom Res, 135, 110167.<br><br>**Aim:** To assess the cost-effectiveness of the DROP (DepRessiOn and Pain) program in primary care patients with comorbid chronic pain and depression using QALYs and clinical outcomes for depression from two perspectives: the health system and society.<br><br>**Design:** Randomised controlled trial	Spain, Catalonia (eight urban primary care centres)	328 patients (167 in the intervention group and 161 in the control group); aged 18-80 years; mean age 60 years; male 17%; female 83%	Major depression and chronic musculoskeletal pain	DROP program	Care as usual	Direct medical care use costs (intervention costs, treatment costs, medication, primary and secondary care costs, primary care emergency, physiotherapy, specialised outpatient care, hospital emergencies, inpatient costs)<br><br>Indirect costs (loss of work productivity costs)	DFDs<br><br>QALYs

**Study details:** Barley, E.A., Walters, P., Haddad, M., Phillips, R., Achilla, E., McCrone, P., Van Marwijk, H., Mann, A. and Tylee, A., 2014. The UPBEAT nurse-delivered personalized care intervention for people with coronary heart disease who report current chest pain and depression: a randomised controlled pilot study. PloS one, 9(6), p.e98704.<br><br>**Aim:** To explore the acceptability and feasibility of procedures for a trial and an intervention, including its potential costs, to inform a definitive randomized controlled trial of nurse-led personalised care intervention for primary care coronary heart disease patients with current chest pain and probable depression<br><br>**Design:** Multicentre randomised controlled trial	UK, South London (17 general practices)	81 patients (41 in the intervention group and 40 in the control group); aged 38-95 years; mean age 65 years; male 65%; female 35%	Depression and coronary heart disease	Personalized care, i.e., UPBEAT	Treatment as usual	Direct medical care use costs (intervention costs, hospital inpatient and outpatient visits, GPs, psychiatrists, psychologists, physiotherapists, counsellors, nurses and other therapists)<br><br>Direct non-medical care use costs (informal care)	QALYs
**Study details**:Basu, R., Ory, M.G., Towne Jr, S.D., Smith, M.L., Hochhalter, A.K. and Ahn, S., 2015. Cost-effectiveness of the chronic disease self-management program: implications for community-based organizations. Frontiers in public health, 3, p.27.<br><br>**Aim:** To perform an economic evaluation of the Chronic Disease Self-Management Program (CDSMP) by utilising a cost-effectiveness analysis of health-related quality of life among CDSMP participants from baseline to 6-month and 12-month follow-up<br><br>**Design:** Pre-post longitudinal design	USA, 17 States (22 organizations)	1,170 individuals; aged 40 years and over; mean age 65 years; male 17%; female 83%	Depression and at least one chronic health condition (which is unclear)	Chronic Disease Self-Management Program (CDSMP)	No intervention	Direct medical care use costs (workshop sessions, trained peer personnel, materials, training space, emergency room visits, hospitalisations)	QALYs

**Study details:** Camacho, E. M., Ntais, D., Coventry, P., Bower, P., Lovell, K., Chew-Graham, C., Baguley, C., Gask, L., Dickens, C. & Davies, L. M. 2016. Long-term cost-effectiveness of collaborative care (vs usual care) for people with depression, comorbid diabetes, or cardiovascular disease: a Markov model informed by the COINCIDE randomised controlled trial. BMJ Open, 6, e012514.<br><br>**Aim:** To evaluate the long-term cost-effectiveness of collaborative care (vs usual care) for treating depression in patients with diabetes or coronary heart disease.<br><br>**Design:** Modelling informed by randomised controlled trial	UK, North West of England (36 primary care (general) practices)	387 patients (191 in the intervention group and 196 in the usual care group); aged ≥18 years; mean age 58 years; male 62%; female 38%	Persistent depressive symptoms, comorbid type 1 or 2 diabetes mellitus or coronary heart disease	Collaborative care	Usual care	Direct medical care use costs (training, primary and community care, hospital inpatient and outpatient care, prescribed medications, private medical expenses)<br><br>Direct non-medical care use costs (travel costs to healthcare appointments)	QALYs
**Study details:** Camacho, E. M., Davies, L. M., Hann, M., Small, N., Bower, P., Chew-Graham, C., Baguely, C., Gask, L., Dickens, C. M., Lovell, K., Waheed, W., Gibbons, C. J. & Coventry, P. 2018. Long-term clinical and cost-effectiveness of collaborative care (versus usual care) for people with mental-physical multimorbidity: cluster-randomised trial. Br J Psychiatry, 213, 456-463.<br><br>**Aim:** To explore the long-term (24-month) effectiveness and cost-effectiveness of collaborative care in people with mental-physical multimorbidity.<br><br>**Design:** Cluster randomised trial	UK, North West of England (36 primary care (general) practices)	387 patients (191 in the intervention group and 196 in the usual care group); aged ≥18 years; mean age 58.5 years; male 62%; female 38%	Persistent depressive symptoms, comorbid type 1 or 2 diabetes mellitus or coronary heart disease	Collaborative care	Usual care	Direct medical care use costs (intervention costs, training, visits to different healthcare professionals, in-patient admission, out-patient, day patient (non-overnight hospital admission), accident and emergency, and primary/ community care)	QALYs

**Study details:** Duarte, A., Walker, J., Walker, S., Richardson, G., Holm Hansen, C., Martin, P., Murray, G., Sculpher, M. & Sharpe, M. 2015. Cost-effectiveness of integrated collaborative care for comorbid major depression in patients with cancer. J Psychosom Res, 79, 465-70.<br><br>**Aim:** To estimate the cost-effectiveness of Depression Care for People with Cancer compared with usual care from a health service perspective<br><br>**Design:** Multicentre randomised controlled trial	UK, Scotland (3 cancer centres and their associated clinics (Glasgow, Edinburgh and Dundee))	500 adults (253 in the intervention group and 247 in the usual care group); aged ≥ 18 years; mean age 56 years; male 10%; female 90%	Comorbid major depression and cancer	Depression Care for People with Cancer (DCPC)	Usual care	Direct medical care use costs (inpatient hospital and hospice stays, accident and emergency attendances, outpatient appointments for cancer treatment, outpatient appointments for psychological treatment, attendance at NHS-funded day hospices, primary care consultations, prescribed medications, e.g. antidepressants, analgesics and anticancer medication)	QALYs
**Study details:** Goorden, M., van der Feltz-Cornelis, C. M., van Steenbergen-Weijenburg, K. M., Horn, E. K., Beekman, A. T. & Hakkaart-van Roijen, L. 2017. Cost-utility of collaborative care for the treatment of comorbid major depressive disorder in outpatients with chronic physical conditions. A randomized controlled trial in the general hospital setting (CC-DIM). Neuropsychiatr Dis Treat, 13, 1881-1893.<br><br>**Aim:** To evaluate the cost-utility of collaborative care for the treatment of comorbid major depressive disorder in chronically ill patients in the outpatient general hospital setting.<br><br>**Design:** Multicentre randomised controlled trial	Netherlands (5 general hospitals in Amsterdam, Almelo, Hengelo, Ede, and Maastricht)	81 patients (42 in the intervention group and 39 in the usual care group); aged >18 years; mean age 58.5 years; male 61%; female 39%	Comorbid major depressive disorder and chronic physical conditions	Collaborative care treatment	Care as usual	Direct medical care use costs (GP consultation, mental health care institute, psychiatrist/psychologist at an outpatient centre or hospital, occupational health care, medical specialist, paramedic care provider, social worker, consultation for alcohol/drugs, alternative treatment, self-help care, admission to part-time day care, psychiatric hospital admission, and medication)<br><br>Direct non-medical care use costs (household work and informal care)	QALYs

**Study details:** Hay, J. W., Katon, W. J., Ell, K., Lee, P. J. & Guterman, J. J. 2012. Cost-effectiveness analysis of collaborative care management of major depression among low-income, predominantly Hispanics with diabetes. Value Health, 15, 249-54.<br><br>**Aim:** To evaluate the cost-effectiveness of a socioculturally adapted collaborative depression care program among low-income Hispanics with diabetes<br><br>**Design:** Randomised controlled trial	USA, Los Angeles County public community clinics	387 patients (193 in the intervention group and 194 in the usual care group); aged ≥18 years; mean age not reported; male 18%; female 82%	Major Depression and diabetes	Multifaceted Diabetes and Depression Program (MDDP)	Enhanced usual care	Direct medical care use costs (medications, laboratory, emergency department, outpatient, inpatient, medical equipment, and additional medical costs not otherwise specified)<br><br>Direct non-medical care use costs (home care)	QALYs
**Study details:** Johnson, J. A., Lier, D. A., Soprovich, A., Al Sayah, F., Qiu, W. & Majumdar, S. R. 2016. Cost-Effectiveness Evaluation of Collaborative Care for Diabetes and Depression in Primary Care. Am J Prev Med, 51, e13-20.<br><br>**Aim:** To present an economic evaluation of a collaborative care model for patients with Type 2 diabetes and depressive symptoms in the Canadian primary care setting<br><br>**Design:** Controlled implementation trial	Canada, Alberta (in four primary care networks)	227 patients (95 in the intervention group, 62 in the enhanced care and 71 in the usual care group), aged ≥18 years; mean age 58 years; male 45%; female 55%	Depressive symptoms and type 2 diabetes	Collaborative care	Enhanced care<br><br>Usual care	Direct medical care use costs (inpatient admissions, outpatient visits, provider visits, mental health services, registered nurse care time and activities, training, physician specialist consultation)	DFDs<br><br>QALYs

**Study details:** Jonkers, C. C. M., Lamers, F., Evers, S., Bosma, H., Metsemakers, J. F. & Van Eijk, J. T. M. 2009. Economic evaluation of a minimal psychological intervention in chronically ill elderly patients with minor or mild to moderate depression.: A randomized trial (the DELTA-study). International Journal of Technology Assessment in Health Care, 25, 497-504.<br><br>**Aim:** To assess, from a societal perspective, the cost-effectiveness of the minimal psychological intervention (MPI) compared with usual care.<br><br>**Design:** Two-armed randomised controlled trial	Netherlands, South of the Netherlands (89 primary care practices)	228 patients (110 in the intervention group and 118 in the usual care group); aged 60 years and over; mean age 69.7 years; male 54%: female 46%	Depression with type 2 diabetes mellitus or chronic obstructive pulmonary disease	Minimal psychological intervention (MPI)	Usual care	Direct medical care use costs (home visits, training for nurses, visits to GP, inpatient and outpatient, allied health professionals such as physiotherapists, dieticians, professional home care, medical devices and assistive devices, medication, intervention costs)<br><br>Direct non-medical care use costs (informal care, nurses' travel expenses)<br><br>Indirect costs (productivity loss costs estimated using the friction cost approach)	DFDs<br><br>QALYs
**Study details:** Katon, W., Unutzer, J., Fan, M. Y., Williams, J. W., Jr., Schoenbaum, M., Lin, E. H. & Hunkeler, E. M. 2006. Cost-effectiveness and net benefit of enhanced treatment of depression for older adults with diabetes and depression. Diabetes Care, 29, 265-70.<br><br>**Aim:** To determine the incremental cost-effectiveness and net benefit of a depression collaborative care program compared with usual care for patients with diabetes and depression.<br><br>**Design:** Randomised controlled trial	USA, 18 primary care clinics from eight healthcare organizations in five states	418 patients (204 in the intervention group and 214 in the usual care group); aged >60 years; mean age 70 years; male 47%; female 53%	Major depression and diabetes	Improving Mood-Promoting Access to Collaborative (IMPACT)	Usual care	Direct medical care use costs (outpatient, medical and mental health care, speciality, urgent care, emergency visits, non-antidepressant prescriptions, laboratory, X-rays, inpatient, medical, mental health treatment, medical/surgical admissions, intervention costs)	DFDs<br><br>QALYs

**Study details:** Katon, W., Russo, J., Lin, E. H., Schmittdiel, J., Ciechanowski, P., Ludman, E., Peterson, D., Young, B. & Von Korff, M. 2012. Cost-effectiveness of a multicondition collaborative care intervention: a randomized controlled trial. Arch Gen Psychiatry, 69, 506-14.<br><br>**Aim:** To evaluate the cost-effectiveness of a multicondition collaborative treatment program (TEAMcare) compared with usual primary care in outpatients with depression and poorly controlled diabetes or coronary heart disease.<br><br>**Design:** Randomised controlled trial	USA, Washington (fourteen primary care clinics of an integrated health care system)	214 patients (106 in the intervention group and 108 in the usual care group); adults (no age range specified); mean age 56.8 years; male 51%; female 49%	Depressive disorder and diabetes or coronary heart disease	TEAMcare	Usual primary care	Direct medical care use costs (outpatient, inpatient, emergency, laboratory, radiology, pharmacy, primary care, speciality care, mental health, ambulatory surgery, alternative health care, dialysis, durable medical equipment, and physical and occupational therapy, intervention costs)	Depression-free days (DFDs)<br><br>Quality-adjusted life years (QALYs)
**Study details:** Kearns, B., Rafia, R., Leaviss, J., Preston, L., Brazier, J. E., Palmer, S. & Ara, R. 2017. The cost-effectiveness of changes to the care pathway used to identify depression and provide treatment amongst people with diabetes in England: a model-based economic evaluation. BMC Health Serv Res, 17, 78.<br><br>**Aim:** To assess the health economic outcomes associated with diabetes and depression and assess the cost-effectiveness of potential policy changes to improve the care pathway: improved opportunistic screening for depression, collaborative care for depression treatment, and the combination of both.<br><br>**Design:** Decision Analytic model	UK, England (primary care)	No age range specified; mean age 66.5 years; no male and female ratio reported	Depression and type-2 diabetes	Policy changes to improve the care pathway:<br><br>1) improved opportunistic screening for depression,<br>2) collaborative care for depression treatment, and<br>3) a combination of both	Improved opportunistic screening<br><br>Current practice<br><br>Combined policy	Direct medical care use costs (GP appointments, psychotherapy sessions, opportunistic screening for depression, antidepressants)<br><br>Direct non-medical care use costs (informal care)<br><br>Indirect costs (productivity losses costs)	QALYs

**Study details:** Moayeri, F., Dunt, D., Hsueh, Y. A. & Doyle, C. 2019. Cost-utility analysis of telephone-based cognitive behaviour therapy in chronic obstructive pulmonary disease (COPD) patients with anxiety and depression comorbidities: an application for willingness to accept concept. Expert Rev Pharmacoecon Outcomes Res, 19, 331-340.<br><br>**Aim:** To assess, from a health service payer perspective, the cost-utility of the telephone-based cognitive behavioural therapy (TB-CBT) compared with a befriending program as a nondirective emotional, social support provided by volunteers, using a willingness to accept (WTA)/ willingness to pay (WTP) disparity concept.<br><br>**Design:** Pragmatic, two-armed randomised control trial	Australia, Melbourne (four tertiary hospitals and pulmonary rehabilitation programs)	110 patients (54 in the intervention group and 56 in the control group); aged 45 years or over; mean age 68 years; male 35%; female 65%	Depression and anxiety comorbidities with chronic obstructive pulmonary disease	Telephone-based cognitive behavioural therapy (TB-CBT) plus current standard care	Current standard care plus placebo-befriending phone calls	Direct medical care use costs (GP visit, specialist visit, allied health care, medical aid and assistant devices, prescribed and over-the-counter medicine, hospital and emergency visit, intervention costs)	QALYs
**Study details:** Nobis, S., Ebert, D. D., Lehr, D., Smit, F., Buntrock, C., Berking, M., Baumeister, H., Snoek, F., Funk, B. & Riper, H. 2018. Web-based intervention for depressive symptoms in adults with types 1 and 2 diabetes mellitus: a health economic evaluation. Br J Psychiatry, 212, 199-206.<br><br>**Aim:** To assess the cost-effectiveness of a web-based intervention (GET.ON M.E.D.) for individuals with diabetes and comorbid depression compared with an active control group receiving web-based psychoeducation<br><br>**Design:** Randomised controlled trial	Germany	260 patients (130 in the intervention group and 130 in the control group), aged 18-79 years; mean age 51 years; male 37%; female 63%	Depressive symptoms and types 1 or 2 diabetes mellitus	GET.ON Mood Enhancer Diabetes (GET.ON M.E.D.)	Web-based psychoeducation	Direct medical care use costs (consultation with a medical practitioner, psychologist, psychotherapists, neurologists, physiotherapy, antidepressants, hospital in-patient, semi-residential rehabilitation, intervention costs)<br><br>Direct non-medical care use costs (travel costs)<br><br>Indirect costs (presenteeism, absenteeism, productivity losses cost based on the human capital approach)	Treatment response<br><br>QALYs

**Study details:** Pan, Y. J., Kuo, K. H., Chan, H. Y. & McCrone, P. 2014. Cost-effectiveness and cost-utility of selective serotonin reuptake inhibitors, serotonin-norepinephrine reuptake inhibitors, and tricyclic antidepressants in depression with comorbid cardiovascular disease. J Psychiatr Res, 54, 70-8.<br><br>**Aim:** To compare the cost-effectiveness and cost-utility between antidepressant categories and to test whether and how the presence of CVD affects the economic evaluations of pharmacological treatments of depression.<br><br>**Design:** Observational (administrative database) study	Taiwan	27,484 patients with cardiovascular disease and depression (total 96,501 patients); aged ≥18 years; mean age 59 years; male 39%; female 61%	Depression and comorbid cardiovascular disease	Three antidepressants:<br>1) Selective serotonin reuptake inhibitors (SSRIs)<br>2) Serotonin-norepinephrine reuptake inhibitors (SNRIs)<br>3) Tricyclic antidepressants (TCAs)	Comparison of three antidepressants	Direct medical care use costs (outpatient services, emergency attendances, and inpatient stays)	Sustained treatment-free status (treatment success rate)<br><br>QALYs
**Study details:** Simon, G. E., Katon, W. J., Lin, E. H., Rutter, C., Manning, W. G., Von Korff, M., Ciechanowski, P., Ludman, E. J. & Young, B. A. 2007. Cost-effectiveness of systematic depression treatment among people with diabetes mellitus. Arch Gen Psychiatry, 64, 65-72.<br><br>**Aim:** To evaluate the incremental cost and cost-effectiveness of a systematic depression treatment program among outpatients with co-occurring diabetes mellitus and depression.<br><br>**Design:** Randomised controlled trial	USA, Western Washington (9 primary care clinics)	329 patients (165 in the intervention group and 164 in the control group), middle-aged to elderly (no age range specified); mean age 57.5 years; female 35%; male 65%	Depressive disorder and diabetes mellitus	Systematic depression treatment program	Usual care	Direct medical care use costs (outpatient depression treatment, antidepressant prescriptions, speciality mental health visits, primary care mental health visits, intervention costs, screening)	DFDs

**Study details:** Strong, V., Waters, R., Hibberd, C., Murray, G., Wall, L., Walker, J., McHugh, G., Walker, A. and Sharpe, M., 2008. Management of depression for people with cancer (SMaRT oncology 1): a randomised trial. The Lancet, 372(9632), pp.40-48.<br><br>**Aim:** To assess the efficacy and cost of a nurse-delivered complex intervention that was designed to treat major depressive disorder in patients who have cancer<br><br>**Design:** Randomised controlled trial	UK, Scotland (regional tertiary NHS cancer centre)	200 patients (101 in the intervention group and 99 in the usual care group), adults; mean age 56.6 years; male 29%; female 71%	Major depressive disorder and cancer	Depression Care for People with Cancer (DCPC)	Usual care	Direct medical care use costs (treatment sessions, nurse time and psychiatrist supervision, psychiatrist time, health-care contacts, e.g., visits to primary-care doctor, antidepressant drugs)	QALYs
**Study details:** Walker, S., Walker, J., Richardson, G., Palmer, S., Wu, Q., Gilbody, S., Martin, P., Hansen, C. H., Sawhney, A., Murray, G., Sculpher, M. & Sharpe, M. 2014. Cost-effectiveness of combining systematic identification and treatment of co-morbid major depression for people with chronic diseases: the example of cancer. Psychol Med, 44, 1451-60.<br><br>**Aim:** To achieve the best estimate of the cost-effectiveness of systematic integrated depression management, including systematic case identification and systematic treatment, when compared with usual practice for patients with major depression attending specialist cancer services by using multiple data sources to supplement the data from SMaRT Oncology-1.<br><br>**Design:** Decision Analytic model	UK/Secondary care	Adult patients (no age range specified) diagnosed with cancer and who had a life expectancy of one year or more; no male and female ratio reported	Co-morbid major depression and cancer	Systematic integrated depression management (includes both case identification and treatment)	Usual practice	Direct medical care use costs (treatment costs, primary care physicians and cancer clinic visits along with in-patient stays and out-patient appointments, medical and psychiatric)	QALYs

Supplementary File 5: Critical appraisal of the included economic evaluation studies (model-based)

The methodological quality of the included model-based economic evaluation studies was assessed using the Philips' Checklist.

Dimension of quality	No.	Questions for critical appraisal	Included studies		
			Camacho et al., 2016	Kearns et al., 2017	Walker et al., 2014
Structure					
S1 Statement of decision problem/objective	1	Is there a clear statement of the decision problem?	Yes	Yes	Yes
	2	Is the objective of the evaluation and model specified and consistent with the stated decision problem?	Yes	Yes	Yes
	3	Is the primary decision maker specified?	No	Yes/No	No
S2 Statement of scope/perspective	4	Is the perspective of the model stated clearly?	Yes	Yes	Yes
	5	Are the model inputs consistent with the stated perspective?	Yes	Yes	Yes
	6	Has the scope of the model been stated and justified?	Yes	Yes	Yes/No
	7	Are the outcomes of the model consistent with the perspective, scope and overall objective of the model?	Yes	Yes	Yes
S3 Rationale for structure	8	Is the structure of the model consistent with a coherent theory of the health condition under evaluation?	Yes	Yes	Yes
	9	Are the sources of data used to develop the structure of the model specified?	Yes	Yes	Yes
	10	Are the causal relationships described by the model structure justified appropriately?	Yes	Yes	Yes
S4 Structural assumptions	11	Are the structural assumptions transparent and justified?	No (Background all-cause mortality assumed to be 0 is not justified. Primary analysis assumed equivalent probabilities/utilities (usual care) for both trial groups is not justified.	Yes	Yes
	12	Are the structural assumptions reasonable given the overall objective, perspective and scope of the model?	Yes	Yes	Yes
S5 Strategies/comparators	13	Is there a clear definition of the options under evaluation?	Yes	Yes	Yes
	14	Have all feasible and practical options been evaluated?	Yes (extrapolation of the findings from a short-term RCT)	Yes	Yes
	15	Is there justification for the exclusion of feasible options?	Not Applicable	No	Not Applicable
S6 Model type	16	Is the chosen model type appropriate given the decision problem and specified causal relationships within the model?	Yes	Yes	Yes
S7 Time horizon	17	Is the time horizon of the model sufficient to reflect all important differences between options?	No	Yes	Yes/No (uncertainty about time horizon

					considered in sensitivity analysis)
	18	Are the time horizon of the model, the duration of treatment and the duration of treatment effect described and justified?	Yes	Yes	Yes
S8 Disease states/pathways	19	Do the disease states (state transition model) or the pathways (decision tree model) reflect the underlying biological process of the disease in question and the impact of interventions?	Yes	Yes	Yes
S9 Cycle length	20	Is the cycle length defined and justified in terms of the natural history of disease?	Yes (to reflect the transition observed during the trial)	Not applicable	No
Data					
D1 Data identification	21	Are the data identification methods transparent and appropriate given the objectives of the model?	Yes	Yes	Yes
	22	Where choices have been made between data sources, are these justified appropriately?	Yes	Yes	Yes
	23	Has particular attention been paid to identifying data for the important parameters in the model?	Yes	Yes	Yes
	24	Has the quality of the data been assessed appropriately?	Yes	Yes	Yes
	25	Where expert opinion has been used, are the methods described and justified?	Not Applicable	No	Not Applicable
D2 Data modelling	26	Is the data modelling methodology based on justifiable statistical and epidemiological techniques?	Not Applicable	Yes	Yes
D2a Baseline data	27	Is the choice of baseline data described and justified?	Yes	Yes	Yes
	28	Are transition probabilities calculated appropriately?	Yes	Not applicable	Yes
	29	Has a half-cycle correction been applied to both cost and outcome?	No	Not applicable	No
	30	If not, has this omission been justified?	No	Not applicable	No
D2b Treatment effects	31	If relative treatment effects have been derived from trial data, have they been synthesised using appropriate techniques?	Yes	No	No
	32	Have the methods and assumptions used to extrapolate short term results to final outcomes been documented and justified?	Yes	Not applicable	No
	33	Have alternative assumptions been explored through sensitivity analysis?	Yes	Yes	Yes
	34	Have assumptions regarding the continuing effect of treatment once treatment is complete been documented and justified?	Yes	No	No
	35	Have alternative assumptions been explored through sensitivity analysis?	Yes	Yes	Yes
D2c Costs	36	Are the costs incorporated into the model justified?	Yes	Yes	Yes

	37	Has the source for all costs been described?	Yes	Yes	Yes
	38	Have discount rates been described and justified given the target decision-maker?	Yes	Yes	Yes
D2d Quality of life weights (utilities)	39	Are the utilities incorporated into the model appropriate?	Yes	Yes	Yes
	40	Is the source for the utility weights referenced?	Yes	Yes	Yes
	41	Are the methods of derivation for the utility weights justified?	No	No	No
D3 Data incorporation	42	Have all data incorporated into the model been described and referenced in sufficient detail?	Yes	Yes	Yes
	43	Has the use of mutually inconsistent data been justified (i.e. are assumptions and choices appropriate)?	Not Applicable	Yes	Yes
	44	Is the process of data incorporation transparent?	Yes	Yes	Yes
	45	If data have been incorporated as distributions, has the choice of distribution for each parameter been described and justified?	No	No	Yes
	46	If data have been incorporated as distributions, is it clear that second order uncertainty is reflected?	Yes	No	No
D4 Assessment of uncertainty	47	Have the four principal types of uncertainty been addressed?	Yes	No	No
	48	If not, has the omission of particular forms of uncertainty been justified?	Not Applicable	No	No
D4a Methodological	49	Have methodological uncertainties been addressed by running alternative versions of the model with different methodological assumptions?	Yes	No	No
D4b Structural	50	Is there evidence that structural uncertainties have been addressed via sensitivity analysis?	Yes	No	No
D4c Heterogeneity	51	Has heterogeneity been dealt with by running the model separately for different subgroups?	Yes	No	No
D4d Parameter	52	Are the methods of assessment of parameter uncertainty appropriate?	Yes	Yes	Yes
	53	If data are incorporated as point estimates, are the ranges used for sensitivity analysis stated clearly and justified?	No	No	No
Consistency					
C1 Internal consistency	54	Is there evidence that the mathematical logic of the model has been tested thoroughly before use?	No	No	No
C2 External consistency	55	Are any counterintuitive results from the model explained and justified?	Not Applicable	Not Applicable	No
	56	If the model has been calibrated against independent data, have any differences been explained and justified?	No	No	No
	57	Have the results of the model been compared with those of previous models and any differences in results explained?	No (comparison with trials/reviews)	No	No

Supplementary File 6: Critical appraisal of the included economic evaluation studies (except modelling studies)

The methodological quality of the included economic evaluation studies (except modelling studies) was assessed using Drummond's Checklist.

Study	Drummond's Checklist Items Number									
	1	2	3	4	5	6	7	8	9	10
Aragonès et al., 2020	Yes (but no mention of alternatives being compared)	Yes	Yes	Yes	Yes	Yes	No (justification given)	Yes	No (only partly)	Can't tell (does not state generalisability)
Barley et al., 2014	No	Yes	Yes	Yes	Yes	Yes	No	No	No (only bootstrapping)	Can't tell (does not state generalisability)
Basu et al., 2015	No (no comparison of alternatives, perspective for analysis)	Yes	Yes	Can't tell (in the absence of perspective for analysis)	Yes	Yes	No	Yes	No	Can't tell (does not state generalisability, implications of uncertainty)
Camacho et al., 2018	Yes	Yes	Yes	Yes	Yes	Yes	No (justification given)	Yes	Yes	Can't tell (does not state generalisability and need for future research)
Duarte et al., 2015	Yes	Yes	Yes	Yes	Yes	Yes	No (justification given)	Yes	Yes	Can't tell (does not state the need for future research)
Goorden et al., 2017	Yes	Yes	Yes	Yes	Yes	Yes	No	Yes	No (only partly)	Can't tell (does not state generalisability)
Hay et al., 2012	Yes	Yes	Yes	Yes	Yes	Yes	No	Yes	No	Can't tell (does not state generalisability, implications of uncertainty)
Johnson et al., 2016	Yes	Yes	Yes	Yes	Yes	Yes	No (justification given)	Yes	Yes	Can't tell (does not state generalisability and need for future research)
Jonkers et al., 2009	Yes	Yes	Yes	Yes	Yes	Yes	Yes	Yes	Yes	Yes

Katon et al., 2006	Yes	Yes	Yes	Can't tell (Perspective not entirely clear)	Yes	Yes	No	Yes	Yes	Can't tell (does not state generalisability)
Katon et al., 2012	Yes	Yes	Yes	Yes	Yes	Yes	No	Yes	Yes	Can't tell (does not state generalisability and need for future research)
Moayeri et al., 2018	Yes	Yes	Yes	Yes	Yes	Yes	No (justification given)	Yes	Yes	Can't tell (does not state generalisability and need for future research)
Nobis et al., 2018	Yes	Yes	Yes	Yes	Yes	Yes	No	Yes	Yes	Can't tell (does not state generalisability)
Pan et al., 2014	Yes	Yes	Yes (using observational data)	Yes	Yes	Yes	No	Yes	Yes	Yes
Simon et al., 2007	Can't tell	Yes	Yes	Yes	Yes	Yes	No	Yes	Yes	Can't tell (does not state generalisability)
Strong et al., 2008	No	Yes	Yes	Yes	Yes	Yes	No	Yes (briefly)	Yes	Yes

Supplementary File 7: Reporting quality assessment of the included economic evaluation studies

The reporting quality of the included studies was assessed using the CHEERS 2022 Checklist.

Item No.	Aragonès et al., 2020	Barley et al., 2014	Basu et al., 2015	Camacho et al., 2016	Camacho et al., 2018	Duarte et al., 2015
1	Title, Page 1 (but interventions being compared not reported)	Not reported	Title, Page 1 (but interventions being compared not reported)	Title, Page 1	Title, Page 1	Title, Page 1 (but interventions being compared not reported)
2	Abstract, Page 1	Abstract, Page 1 (but lacked key information)	Not reported	Abstract, Page 1 (but lacked key information)	Abstract, Page 1 (but lacked key information)	Abstract, Page 1
3	Introduction, Last two paragraphs	Not reported	Introduction, Last paragraph	Introduction, Last two paragraphs	Introduction	Introduction, Last two paragraphs
4	Not reported	Not reported	Not reported	Not reported	Not reported	Not reported
5	Methods, First paragraph (design overview)	Methods, Participants	Materials and Methods, First paragraph	Methods, First paragraph	Methods (Trial design and participants last paragraph)	Methods, First paragraph
6	Methods, First Paragraph	Methods, Study setting	Materials and Methods, First paragraph	Methods, First paragraph	Methods, Second paragraph	Methods, First paragraph
7	Methods, Third Paragraph	Methods, Intervention, Control	Materials and Methods, Ninth Paragraph	Methods, First paragraph	Methods, First paragraph	Methods, Third Paragraph
8	Methods (Data analyses), Fourth Paragraph	Methods, Costs of personalised care (PC)	Not reported	Methods (Economic model), First paragraph	Methods (Outcomes), Second Paragraph	Methods (Analysis), First Paragraph
9	Methods (Intervention), First Paragraph	Methods, Measurement	Materials and Methods (Study sample) paragraph	Methods, Economic model, Second Paragraph	Methods, Outcomes, Second Paragraph	Methods (Analysis), First Paragraph; No reason reported for appropriateness
10	Methods (Data analyses), First Paragraph	Not reported	Not reported	Methods, Economic model, Second Paragraph	Methods, Outcomes, Second Paragraph	Methods (Resource use and costs), Last Paragraph
11	Methods (Utility), First Paragraph	Methods, Outcomes	Materials and Methods (Measures) paragraph	Methods, Measuring health benefit Paragraph	Methods, Outcomes First and Fourth Paragraphs	Methods, Outcomes section

12	Methods (Utility), First Paragraph	Methods, Outcomes, Costs of PC (first sentence)	Materials and Methods (Measures) paragraph	Methods, Measuring health benefit Paragraph	Methods, Outcomes First and Fourth Paragraphs	Methods, Outcomes section
13	Methods (Utility), First Paragraph	Methods, Outcomes, Costs of PC (first sentence)	Materials and Methods (Analysis) HRQOL, EQ-5D, and QALYs paragraph	Methods, Measuring health benefit Paragraph	Methods, Outcomes First and Fourth Paragraphs	Methods, Outcomes section
14	Methods (Costs), First and Second Paragraph	Methods, Costs of PC	Materials and Methods, Cost measures paragraph	Methods, Measuring costs Paragraph	Methods, Outcomes Second and Third Paragraphs	Methods, Resource use and costs section
15	Methods (Costs), First and Second Paragraph; Table 2	Methods, Costs of PC	Materials and Methods, Cost measures paragraph	Methods, Measuring costs Paragraph	Methods, Outcomes, Third Paragraphs	Methods, Resource use and costs section
16	Not applicable	Not applicable	Not applicable	Methods, Economic model Paragraph	Not applicable	Not applicable
17	Methods (Section 2.7 and 2.8)	Methods, Statistical analyses (Fourth and Fifth Paragraphs)	Materials and Methods, Analysis section	Methods, Economic model Paragraph	Methods, Statistical analysis Section	Methods, Analysis section
18	Not reported	Not reported	Not reported	Online supplementary table S2	Not reported	Not reported
19	Not reported	Not reported	Not reported	Not reported	Not reported	Not reported
20	Methods (Data analysis), Second Paragraph	Methods, Statistical analyses (Fifth Paragraph)	Not reported	Methods, Last Two Paragraphs	Methods, Statistical analysis, Last Paragraph	Methods (Analysis), Third, Fourth and Fifth Paragraphs
21	Not reported	Not reported	Not reported	Not reported	Not reported	Not reported
22	Not applicable	Not applicable	Not applicable	Table 1	Not applicable	Not applicable
23	Results, Sections (3.2 and 3.3), Tables (2 and 3)	Results, QALY gains, Appendix (S3, S4, S5), Figure 3 (incremental cost not reported)	Results, Fourth Paragraph and Table 4	Results, Economic model Paragraph and Table 2, Figure 2	Results, Cost-effectiveness Section, Tables 2 and 3, Figure 1	Results, Cost-effectiveness analysis Paragraph, Tables 2-4
24	Results, Section 3.4 and Table 3	Not reported	Not reported	Results, Last Two Paragraphs, Table 2, Figures 2 and 3	Tables 2 and 3, Figure 1	Results, Cost-effectiveness analysis Paragraph, Table 4, Figure 1

25	Not reported	Not reported	Not reported	Not reported	Not reported	Not reported
26	Discussion (No reporting on generalisability)	Discussion, Potential costs of PC (no reporting on limitations, ethical consideration and how these could affect patients, policy, or practice)	Discussion (No reporting on generalisability)	Discussion	Discussion (no reporting on generalisability and future research direction)	Discussion (no reporting on future research direction)
27	End of manuscript	Page 1	Not reported	End of manuscript	End of manuscript	Page 1
28	End of manuscript	Page 1	End of manuscript	End of manuscript	Page 1	End of manuscript

Continue...

Item No.	Goorden et al., 2017	Hay et al., 2012	Johnson et al., 2016	Jonkers et al., 2009	Katon et al., 2006	Katon et al., 2012
1	Title, Page 1 (but interventions being compared not reported)	Title, Page 1 (but interventions being compared not reported)	Title, Page 1 (but interventions being compared not reported)	Title, Page 1 (but interventions being compared not reported)	Title, Page 1 (but interventions being compared not reported)	Title, Page 1 (but interventions being compared not reported)
2	Abstract, Page 1 (but lacked key information)	Abstract, Page 1 (but lacked key information)	Abstract, Page 1 (but lacked key information)	Abstract, Page 1 (but lacked key information)	Abstract, Page 1 (but lacked key information)	Abstract, Page 1 (but lacked key information)
3	Introduction, Last two paragraphs	Introduction, Last paragraph	Introduction, Last two paragraphs	Introduction, Last two paragraphs	Introduction, Last paragraph	Introduction, Last paragraph
4	Not reported	Not reported	Not reported	Not reported	Not reported	Not reported
5	Patients and methods, Third and fourth paragraphs	Methods, First paragraph	Methods, Third paragraph	Methods, Second paragraph	Research design and methods, Second paragraph	Methods, Third paragraph
6	Patients and methods, First paragraph	Methods, First paragraph	Methods, Third paragraph	Methods, Second paragraph	Research design and methods, First paragraph	Methods, Second paragraph
7	Patients and methods, Sixth Paragraph	Methods, First paragraph	Methods, Sixth paragraph	Methods, Fourth paragraph	Research design and methods, Third and Fourth paragraphs	Methods, Third and Fourth paragraphs

8	Patients and methods (Statistical analyses), First Paragraph	Methods (Statistical methods), First Paragraph	Methods, Second Paragraph	Methods, First Paragraph	Not reported	Methods (Patient-level outcomes), Seventh Paragraph
9	Patients and methods (Measures), First Paragraph	Methods (Data Collection), First Paragraph	Methods, Second Paragraph	Methods, First Paragraph	Research design and methods, Statistical analysis, First paragraph	Methods (Patient-level outcomes), First Paragraph
10	Not reported	Not reported	Methods, Second Paragraph	Methods, Measurements (Costs) Last Paragraph	Not reported	Not reported
11	Patients and methods, Measures (Quality of life) section	Methods (Data Collection), Second Paragraph	Methods (Measures), Last Paragraph	Methods, Measurements (Effects) Section	Research design and methods, Outcome measures, First Paragraph	Methods (Patient-level outcomes), Second Paragraph
12	Patients and methods, Measures (Quality of life) section	Methods (Data Collection), Second Paragraph	Methods (Measures), Last Paragraph	Methods, Measurements (Effects) Section	Research design and methods, Outcome measures, Seventh Paragraph	Methods (Patient-level outcomes), Second Paragraph
13	Patients and methods, Measures (Quality of life) section	Methods (Data Collection), Second Paragraph	Methods (Measures), Last Paragraph	Methods, Measurements (Effects) Section	Not reported	Not reported
14	Patients and methods, Measures (Healthcare utilisation costs) section	Methods (Data Collection), Third and Fourth Paragraphs	Methods (Measures), Third and Fourth Paragraphs	Methods, Measurements (Costs) Section	Research design and methods, Outcome measures, Second to Sixth Paragraphs	Methods (Patient-level outcomes), Seven, Eight and Eleven Paragraphs
15	Patients and methods, Measures (Healthcare utilisation costs) section, Last Paragraph	Methods (Data Collection), Third and Fourth Paragraphs	Methods (Measures), Third and Fourth Paragraphs	Methods, Measurements (Costs), Last Paragraph	Research design and methods, Outcome measures, Third Paragraph. Price year not reported	Price year and conversion not reported
16	Not applicable	Not applicable	Not applicable	Not applicable	Not applicable	Not applicable
17	Patients and methods, Statistical analyses section	Methods, Statistical methods section	Methods, Statistical analysis section	Methods, Analyses Section	Research design and methods, Statistical analysis	Methods, Patient-level outcomes section
18	Not reported	Not reported	Not reported	Not reported	Not reported	Not reported

19	Not reported	Not reported	Not reported	Not reported	Not reported	Not reported
20	Patients and methods, Statistical analyses section	Results, Fifth Paragraph	Methods (Statistical analysis), Third and Fourth Paragraphs	Methods, Analyses, Cost-utility analysis, First and Second Paragraph	Research design and methods, Statistical analysis, Last Paragraph	Methods (Patient-level outcomes), Eleventh Paragraphs
21	Not reported	Not reported	Not reported	Not reported	Not reported	Not reported
22	Not applicable	Not applicable	Not applicable	Not applicable	Not applicable	Not applicable
23	Tables 3-6, Figures 3 and 4	Results, Fourth Paragraph, Tables 1-4	Results, Tables 1 and 2	Results, Cost-utility section, Tables 2 and 3	Results, Table 2	Results, Tables 2-4
24	Results, Sensitivity analysis section, Figures 3- 5	Results, Fifth Paragraph, Figure 1	Results, Fifth and Last Paragraphs, Figure 1	Results, Last Paragraph, Table 3, Figure 1	Results, Last Paragraph	Results, Eighth and ninth Paragraphs, Table 3
25	Not reported	Not reported	Not reported	Not reported	Not reported	Not reported
26	Discussion (No reporting on generalisability)	Discussion (No reporting on generalisability)	Discussion (no reporting on future research direction and generalisability)	Discussion	Conclusion (no reporting on generalisability)	Comment (no reporting on future research direction and generalisability)
27	End of a manuscript (Acknowledgement)	End of a manuscript (Acknowledgement)	End of a manuscript	Page 1	End of manuscript	End of a manuscript
28	End of a manuscript	Page 1	End of a manuscript	Not reported	Page 1	End of a manuscript

Continue...

Item No.	Kearns et al., 2017	Moayeri et al., 2018	Nobis et al., 2018	Pan et al., 2014	Simon et al., 2007	Strong et al., 2008	Walker et al., 2014
1	Title, Page 1 (but interventions being compared not reported)	Title, Page 1 (but interventions being compared not reported)	Title, Page 1 (but interventions being compared not reported)	Title, Page 1	Title, Page 1 (but interventions being compared not reported)	Not reported	Title, Page 1 (but interventions being compared not reported)
2	Abstract, Page 1 (but lacked key information)	Abstract, Page 1 (but lacked key information)	Abstract, Page 1 (but lacked key information)	Abstract, Page 1 (but lacked key information)	Abstract, Page 1 (but lacked key information)	Abstract, Page 1 (but lacked key information)	Abstract, Page 1 (but lacked key information)

3	Introduction, Last two paragraphs	Introduction, Last two paragraphs	Introduction, Last two paragraphs	Introduction, Last two paragraphs	Introduction, Last two paragraphs	Not reported	Introduction, Last two paragraphs
4	Not reported	Not reported	Not reported	Not reported	Not reported	Not reported	Not reported
5	Methods, Third paragraph	Methods, First Paragraph	Methods, First Paragraph	Materials and methods, Second paragraph	Methods, Third paragraph	Methods, First, Second and Third Paragraph	Methods, Second Paragraph
6	Methods, First Paragraph	Methods, First Paragraph	Methods, First Paragraph	Materials and methods, First Paragraph	Methods, Second paragraph	Methods, First Paragraph	Methods, First Paragraph
7	Methods, First Paragraph	Methods, First Paragraph	Introduction, Last Paragraph	Materials and methods, Section 2.8, Second paragraph	Methods, Fourth Paragraph	Methods, Fourth paragraph	Methods, First Paragraph
8	Methods (Assessment of cost-effectiveness), First Paragraph	Methods, First Paragraph	Methods (Measuring resource use), First Paragraph	Introduction, Last Paragraph	Comment, Fifth Paragraph	Not reported	Methods (Costs and outcomes), Paragraph
9	Methods (Assessment of cost-effectiveness), Second Paragraph	Methods, Section 2.2, First Paragraph	Methods, Outcome measures, First Paragraph	Materials and methods, Section 2.4 Paragraph	Methods, Ninth Paragraph	Methods, Statistical Analysis, Last Paragraph	Methods (Model Structure), First Paragraph
10	Methods (Assessment of cost-effectiveness), Second Paragraph	Methods, Section 2.2, First Paragraph	Not reported	Not reported	Not reported	Methods, Statistical Analysis, Last Paragraph	Methods, Economic Analysis Paragraph
11	Methods, Health-related quality of life and costs	Methods, Section 2.2, Last Paragraph	Methods, Outcome measures, Second paragraph	Materials and methods, Section 2.6	Methods, Ninth Paragraph	Methods, Outcome measures section	Methods (Design), Paragraph
12	Methods, Health-related quality of life and costs	Methods, Section 2.2, Last Paragraph	Methods, Outcome measures, Second paragraph	Materials and methods, Section 2.5 and 2.6	Methods, Ninth Paragraph	Methods, Outcome measures, Third Paragraph	Methods (Costs and outcomes), Paragraph
13	Methods, Health-related quality of life and costs	Not reported	Methods, Outcome measures, Second paragraph	Materials and methods, Section 2.5 and 2.6	Methods, Ninth Paragraph	Methods, Outcome measures section	Not reported

14	Methods, Health-related quality of life and costs	Methods, Section 2.2, Health-care utilization and costs	Methods, Measuring resource use section	Materials and methods, Section 2.8, First Paragraph	Methods, Tenth Paragraph	Methods, Statistical Analysis, Last Paragraph	Methods (Costs and outcomes), Paragraph, Data sources
15	Methods (Assessment of cost-effectiveness), Second Paragraph	Methods, Section 2.2, Health-care utilization and costs, Second Paragraph	Methods, Measuring resource use, First Paragraph	Materials and methods, Section 2.8, First Paragraph	Methods, Last Paragraph, Price year not reported	Methods, Statistical Analysis, Last Paragraph	Methods (Costs and outcomes), Paragraph
16	Methods, Model structure and Model inputs, Figure 2	Not applicable	Not applicable	Not applicable	Not applicable	Not applicable	Methods, Model Structure
17	Methods, Table 1	Methods, Section 2.4, First and Second Paragraphs	Methods, Analysis of costs, Analysis of cost-effectiveness and cost-utility	Materials and methods, Section 2.9	Methods, Twelfth Paragraph	Methods, Statistical Analysis	Methods, Analysis
18	Not reported	Not reported	Not reported	Not reported	Not reported	Not reported	Not reported
19	Not reported	Not reported	Not reported	Not reported	Not reported	Not reported	Not reported
20	Methods, Assessment of uncertainty	Methods, Section 2.4, Last Paragraph	Methods, Sensitivity analyses	Materials and methods, Section 2.10	Methods, Ninth Paragraph	Methods, Statistical Analysis, Second Paragraph	Methods, Analysis (Sensitivity and scenario analysis)
21	Not reported	Not reported	Not reported	Not reported	Not reported	Not reported	Not reported
22	Methods, Model inputs, First Paragraph, Table 1	Not applicable	Not applicable	Not applicable	Not applicable	Not applicable	Results, First Paragraph, Table 1
23	Results, Health economic outcomes, Table 4	Results, Tables 2-4, Figure 1	Results, Tables 1 and 2, Figures 1 and 2	Results, Section 3.4, Tables 2 and 3	Results, Tables 1-4	Results, Eleventh Paragraph	Results, First and Second Paragraphs, Table 2
24	Results, Incremental cost-effectiveness ratio, Second Paragraph, Table 4	Results, Tables 4, Figure 2	Results, Sensitivity analyses, Table 2, Figure 2	Results, Section 3.5, Figures 1 and 2	Results, Sixth Paragraph, Figure 2	Results, Eleventh Paragraph	Results, Third, Fourth and Fifth Paragraphs, Table 2
25	Not reported	Not reported	Not reported	Not reported	Not reported	Not reported	Not reported
26	Discussion	Discussion (no reporting on future research direction and generalisability)	Discussion (no reporting on generalisability)	Discussion	Comment (no reporting on generalisability)	Discussion	Discussion
27	End of manuscript	End of manuscript	End of manuscript	End of manuscript	End of manuscript	Page 1	End of manuscript

28	End of manuscript	End of manuscript	Page 1	End of manuscript	End of manuscript	End of manuscript	End of manuscript

Item Descriptions of CHEERS 2022 Checklist

1. Identify the study as an economic evaluation and specify the interventions being compared.
2. Provide a structured summary that highlights context, key methods, results, and alternative analyses.
3. Give the context for the study, the study question, and its practical relevance for decision making in policy or practice.
4. Indicate whether a health economic analysis plan was developed and where available.
5. Describe characteristics of the study population (such as age range, demographics, socioeconomic, or clinical characteristics).
6. Provide relevant contextual information that may influence findings.
7. Describe the interventions or strategies being compared and why chosen.
8. State the perspective(s) adopted by the study and why chosen.
9. State the time horizon for the study and why appropriate.
10. Report the discount rate(s) and reason chosen.
11. Describe what outcomes were used as the measure(s) of benefit(s) and harm(s).
12. Describe how outcomes used to capture benefit(s) and harm(s) were measured.
13. Describe the population and methods used to measure and value outcomes.
14. Describe how costs were valued.
15. Report the dates of the estimated resource quantities and unit costs, plus the currency and year of conversion.
16. If modelling is used, describe in detail and why used. Report if the model is publicly available and where it can be accessed.
17. Describe any methods for analysing or statistically transforming data, any extrapolation methods, and approaches for validating any model used.
18. Describe any methods used for estimating how the results of the study vary for subgroups.
19. Describe how impacts are distributed across different individuals or adjustments made to reflect priority populations.
20. Describe methods to characterise any sources of uncertainty in the analysis.
21. Describe any approaches to engage patients or service recipients, the general public, communities, or stakeholders (such as clinicians or payers) in the design of the study.
22. Report all analytic inputs (such as values, ranges, references) including uncertainty or distributional assumptions.
23. Report the mean values for the main categories of costs and outcomes of interest and summarise them in the most appropriate overall measure.
24. Describe how uncertainty about analytic judgments, inputs, or projections affect findings. Report the effect of choice of discount rate and time horizon, if applicable.
25. Report on any difference patient/service recipient, general public, community, or stakeholder involvement made to the approach or findings of the study
26. Report key findings, limitations, ethical or equity considerations not captured, and how these could affect patients, policy, or practice.
27. Describe how the study was funded and any role of the funder in the identification, design, conduct, and reporting of the analysis
28. Report authors conflicts of interest according to journal or International Committee of Medical Journal Editors requirements.

Supplementary File 8: Interventions components

References	Intervention	Important elements/ Key features	Types of interventions	Level of healthcare provision
Basu et al., 2015	Chronic Disease Self-Management Program (CDSMP)	Deliver CDSMP workshops through licenced sites<br>Workshops were supported by various federal, state and local sources, healthcare organisations and community agencies<br>Recruited people for workshops through referrals from organisations serving older adults (e.g., senior centres, healthcare facilities, and social service organisations, as well as self-referrals from other recruitment activities, including flyers, brochures, and health fairs)<br>Focus on content areas including:<br>1) techniques to manage typical responses to chronic health problems such as frustration, fatigue, pain, and isolation;<br>2) improving healthy behaviour such as physical exercise for maintaining and improving strength, flexibility, and endurance; and<br>3) appropriate use of medications, effective communication with healthcare professionals	Self-management	Predominantly patient-level intervention
Jonkers et al., 2009	Minimal Psychological Intervention (MPI)	Delivered at the patient's home by primary care nurses, who were trained in the Depression in Elderly with Long-Term Afflictions (DELTA) intervention but had not received additional training for type-2 diabetes mellitus or chronic obstructive pulmonary disease<br>MPI is based on principles of cognitive behavioural therapy and self-management<br>DELTA intervention consists of five phases:<br>1) Nurse explores the patient's feelings, cognitions, and behaviours;<br>2) Patient keeps a diary in which they record symptoms, complaints, thoughts, worries, and related feelings and behaviours;<br>3) Patient is challenged to link their mood to the consequent behaviour, using information from the diary;<br>4) Introduce a self-management approach, where the patient explores possibilities to alter their behaviour and where they draw up an action plan; and<br>5) Evaluation of the degree to which goals from the action plan have been achieved<br><br>Intervention is tailor-made, and a home visit could comprise one or more phases<br>Patients received two to ten visits for at most three months, depending on the patient's progress<br>Mean number of visits was four, with a mean duration of 61 minutes	Self-management	Predominantly patient-level intervention

Moayeri et al., 2018	Telephone-based cognitive behavioural therapy (TB-CBT)	1) Initial getting-to-know-you session<br>2) Eight scheduled weekly telephone calls of approximately 30 minutes in length of CBT<br>3) Specific topics of the eight therapy sessions were: session 1, “depression and activity tracking”; session 2, “activity scheduling”; session 3, “relaxation skills”; session 4, “cognitive restructuring”; session 5, “problem-solving”; session 6 “sleep management”; session 7 “review and practice coping skills”; and session 8 “maintaining gains and goodbye”.<br>4) CBT sessions delivered by up to 10 registered or provisionally registered psychologists experienced in telephone CBT and with knowledge of COPD education<br>5) Integrity of interventions and consistency of treatment across sites were maintained with the use of a treatment protocol, therapist competency audits, training workshops for therapists, and ongoing supervision by a clinical psychologist<br>6) CBT telephone calls audio recorded to assist with such monitoring<br>7) Two follow-up assessments using assessment tools (postintervention assessment and second follow-up assessment eight weeks after the CBT intervention)	Telephone-based cognitive behavioural therapy	Predominantly patient-level intervention
Nobis et al., 2018	Web-based intervention, i.e., GET.ON Mood Enhancer Diabetes (GET.ON M.E.D.)	A guided self-help intervention<br>1) Consisted of six minimally guided online sessions, two optional sessions (addressing overweight and healthy sleep) and an optional booster session after four weeks)<br>2) Based on cognitive–behavioural therapy (CBT) (systematic behavioural activation (Cuijpers et al. 2007a) and problem-solving (Cuijpers et al. 2007b))<br>3) Included homework assignments and an online mood diary<br>4) Each session contained diabetes-specific themes<br>5) Participants were supported by a coach (graduate students or psychologists) who provided personalised written feedback (approximately 350 words) within 48 h after receiving the homework<br>6) Communication between the participants and the coaches took place in an asynchronous way via the internal messaging function on the GET.ON M.E.D. platform<br>7) Each coach was supervised by an experienced clinical psychologist	Self-management	Predominantly patient-level intervention
Pan et al., 2014	Three antidepressants:<br>1) Selective serotonin reuptake inhibitors (SSRIs)<br>2) Serotonin-norepinephrine reuptake inhibitors (SNRIs)<br>3) Tricyclic antidepressants (TCAs)	Prescribed at least one antidepressant of interest (SSRIs, SNRIs, and TCAs) for treatment of a major depressive disorder or other depression in 2003	Antidepressants treatment	Predominantly patient-level intervention

Aragonès et al., 2020	DepRessiOn and Pain (DROP)	Based on the chronic care model (Rothman et al. 2003)<br>Included the following main components:<br>1) Optimised management of major depression<br>- Designed to promote and facilitate the optimized management of depression based on algorithms and recommendations drawn from a computerized clinical guideline integrated into the electronic primary care medical record system<br>- Guides GPs in making decisions on diagnosis, treatment, and monitoring of major depression; systems for recording and retrieving information on a patient's clinical status; and automated alerts for clinical situations showing poor control of the illness or risk factors<br>2) Care management<br>- Care manager (psychologist) supports and collaborates with the treating physician in managing the patient<br>- Care manager provides patients with close follow-up support through regular telephone contact (once a month for the first three months and every three months up to a year); follows structured points addressing monitoring of symptoms and personal functioning, adherence to treatment, and therapeutic advice;<br>3) Psychoeducational intervention programme for patients with chronic pain and depression<br>- Care manager led group psychoeducational sessions to help patients better understand pain and depression and encourage them to take an active role in managing their conditions<br>- Nine 2-hour sessions held once a week<br>- Content of the psychoeducational sessions covers the following areas: understanding pain; managing emotions; basic relaxation techniques; cognitive restructuring strategies; problem-solving; establishment of life goals; relationships between pain and physical activity, healthy postures, and sleep; maintenance of the strategies learned; and preparation of plans to be applied in the event of temporary setbacks.<br>- In order to promote the active and independent role of the patient, "homework" is assigned after each session, which will be reviewed at the beginning of the following session	Collaborative care	Predominantly organisational-level intervention

Barley et al., 2014	Personalized care, i.e., UPBEAT	Nurse-delivered intervention<br>Nurse act as a case manager and conducts a standardised, face-to-face, biopsychosocial assessment (including physical and mental health, difficulties with current treatment regimens, problems with daily activities and social problems).<br>Patients are then helped to identify up to three problems that they consider contributing to their depression and which they most want to address.<br>The nurse-case managers provide information, sign-post patients to existing resources (e.g. leisure centres, social clubs, Improving Access to Psychological Therapy (IAPT) services) and use evidence-based behaviour change techniques to help patients set and achieve goals.<br>The underlying intention of the intervention is to increase the patient's self-efficacy to achieve their desired goals (as opposed to goals determined by others, such as symptom management or reduction of cardiac risk factors).<br>Details of the assessment and action plan were recorded in a 'personalised health plan' that the patient holds.<br>Follow-up interviews were conducted via telephone to determine progress and/or set new goals.<br>Calls were planned to last 15 minutes and were scheduled weekly initially and then at increasing intervals according to patient need.<br>During the 6-month intervention period, weekly meetings were held with research team clinicians (a GP academic and two psychiatrists) to ensure fidelity to the intervention.	Self-management	Predominantly organisational-level intervention
Camacho et al., 2016	Collaborative care	Choice of appropriate evidence-based low-intensity psychological treatments/interventions<br>Delivered over three months through Improving Access to Psychological Therapy (IAPT) services<br>Case management is provided jointly by the practice nurse and a Psychological Well Being Practitioner (PWP)	Collaborative care	Predominantly organisational-level intervention
Camacho et al., 2018	Collaborative care	Integrated physical and mental healthcare<br>Received up to eight face-to-face sessions of brief psychological therapy delivered by a case manager over three months<br>Case managers = PWPs employed by IAPT services<br>PWPs and practice nurses delivered care to participants<br>First session lasted for 45 minutes, during which the PWP identified links between participants' mood and management of their long-term conditions to formulate a problem statement.<br>Subsequent treatment sessions were scheduled to last for 30–40 min, and participants could choose to engage in behavioural activation, graded exposure, cognitive restructuring and/or lifestyle change<br>A 10 min collaborative meeting (by telephone or in-person) between the participant, PWP and a practice nurse from the participant's general practice was scheduled to take place during treatment sessions two and eight to facilitate the integration of care<br>Collaborative meetings focused on ensuring that psychological treatments did not complicate current management, reviewing patients' progress, reviewing relevant physical and mental health outcomes and planning future care.<br>The final session also included education about relapse prevention strategies<br>PWPs expected to liaise with the practice nurse and participants' GPs about medication and update on participant progress.	Collaborative care	Predominantly organisational-level intervention

Duarte et al., 2015	Depression Care for People with Cancer (DCPC)	An intensive, multicomponent, manualised treatment programme that integrates specialist depression management with both cancer treatment and primary care<br>Systematically delivered by a team that comprises specially trained cancer nurses and supervising psychiatrists working in collaboration with the patient's oncology team and primary care physician<br>Nurses establish a therapeutic relationship with the patients, provide information about depression and its treatment, deliver brief evidence-based psychological interventions (problem-solving therapy and behavioural activation) and monitor patients' progress.<br>Psychiatrists supervise treatment, aiming to achieve and maintain treatment targets, advise primary care physicians about prescribing antidepressants, and provide direct consultations to patients who are not improving<br>Initial treatment phase comprises a maximum of ten sessions with the nurse (at a cancer or primary care clinic, or if necessary, by telephone) over four months.<br>After this initial treatment period, patients' progress is monitored monthly by telephone (through an automated system supplemented by nurse calls) for a further eight months; additional sessions with the nurse are provided for patients not meeting treatment targets.<br>Nurse-delivered intervention at the centre over an average of seven sessions	Collaborative care	Predominantly organisational-level intervention
Goorden et al., 2017	Collaborative care	Treatment is provided by a team consisting of the patient, the Consultant Psychiatric Nurse (CPN) (care manager), and the Consultation-Liaison (CL) psychiatrist at the outpatient clinic of the general hospital<br><br>Included:<br>1) Guided self-help and problem-solving treatment provided by the CPN in a one-to-one session;<br>2) Antidepressants prescribed by the CL psychiatrist according to an algorithm and monitored by a web-based tracking system that functioned as a supportive decision aid for the CPN care manager; and<br>3) Consultations with the CL psychiatrist if necessary<br><br>Treatment response was monitored biweekly with the Patient Health Questionnaire (PHQ)-9	Collaborative care	Predominantly organisational-level intervention
Hay et al., 2012	Multifaceted Diabetes and Depression Program (MDDP)	Key elements include:<br>1) Problem-solving therapy provided by Diabetes Depression Clinical Specialists (DDCS) and/or antidepressant medications prescribed by the treating Primary Care Provider (PCP);<br>2) DDCS monthly telephone follow-up symptom monitoring, treatment maintenance, and relapse prevention; and<br>3) Care and service system navigation by the DDCS and an assistant patient navigator.<br>4) A psychiatrist and principal investigator of the study provided weekly telephone DDCS supervision and, if requested, the psychiatrist provided PCP antidepressant medication telephone consultation.	Collaborative care	Predominantly organisational-level intervention

Johnson et al., 2016	Collaborative care	Nurse-led<br>Implemented in the Canadian primary care setting<br>Adapted from the TEAMCare approach (Katon et al. 2010)<br>Key elements:<br>1) a registered nurse care manager (CM) coordinated collaborative team management.<br>- CM worked with the patient to develop a shared care plan, offered support and problem-solving techniques to optimize self-management, and closely monitored treatment adherence and outcomes;<br>- CM provided active in-person or telephone follow-ups once or twice per month at their discretion to reassess symptoms and assist patients in achieving goals;<br>- CM consulted with psychiatrists or endocrinologists regularly to review new cases and ongoing patient progress and discuss management recommendations based on locally developed and endorsed evidence-based care algorithms<br>- The CM communicated recommendations to family physicians, who remained responsible for all final treatment decisions and all prescriptions<br>- Management of depressive symptoms involved using antidepressant medication, psychotherapy, or both.<br>- Once patients achieved symptom amelioration (PHQ o10), a relapse prevention plan was developed while continuing to work toward cardiometabolic control and lifestyle modifications	Collaborative care	Predominantly organisational-level intervention
Katon et al., 2006	Improving Mood-Promoting Access to Collaborative (IMPACT)	Delivered by a trained Depression Care Manager (DCM)- in most organizations, this was a nurse<br>DCMs received initial training on pharmacotherapy and PST-PC during a 2-day workshop and were required to complete at least five videotaped training cases of PST-PC supervised by a psychologist.<br>DCM provided a behavioural activation intervention to all patients (i.e., structured, positive activities like exercise) and an initial choice of Problem-Solving Treatment developed for Primary Care (PST-PC) or enhanced treatment with antidepressant medication prescribed by the primary care physician<br>PST-PC is a six- to eight-session manualized psychotherapy program<br>DCMs received weekly supervision by a psychiatrist and primary care physician with geriatric expertise in order to monitor the progress of treatment and adjust treatment plans based on clinical response<br>Initial medication treatment would be augmented with PST-PC based on partial or nonresponse and vice versa<br>DCMs followed patients in person or by telephone approximately every two weeks over the acute treatment phase (3–6 months) and approximately once a month in the continuation phase (6–12 months)	Collaborative care	Predominantly organisational-level intervention

Katon et al., 2012	TEAMcare	Patient-centred, team-based collaborative care management intervention for patients with multiple chronic conditions<br>Used a combination of principles from collaborative care depression interventions (Gilbody et al. 2006) and the chronic care model (Wagner et al. 2001) and integrated a treat-to-target medication strategy initially developed for diabetes (Riddle et al. 2003).<br>One consistent treatment approach was applied systematically across three chronic illnesses (diabetes, depression, and coronary heart disease)<br>A physician-supervised nurse care manager was added to the primary care team to enhance patient self-management, treatment intensification, coordination, and continuity of care<br>Nurse care manager worked closely with each patient's Primary Care Physicians (PCPs) to optimize the systematic management of chronic illnesses<br>Nurse care managers worked with patients and PCPs to identify clinical goals and develop individualized care plans<br>Nurse educated patients and used behavioural activation, motivational interviewing, and problem-solving strategies to help patients perform specific self-care activities (i.e., self-monitoring of BP and improving adherence to medication, diet, and exercise regimens)<br>Nurse tracked patient progress using a care management electronic information system and reviewed their caseloads weekly with a consulting psychiatrist and internist or family physician<br>Care managers communicated treatment recommendations based on the physician caseload review and treat-to-target algorithms to the PCPs<br>Weekly systematic case reviews with physician consultants<br>Nurse care managers proactively monitored patients with visits or telephone calls (initially 2-3 contacts a month), administered the Patient Health Questionnaire-9 depression questionnaire, and reviewed home BP or<br>Glucose control and laboratory test results.<br>Frequency of later contacts depended on clinical response.<br>Once patients achieved clinical targets (depression, HbA1c, SBP, and LDL-C), they worked with care managers to formulate a maintenance plan for follow-up with their primary care team.<br>During the maintenance phase, care managers followed up with the patients with telephone calls every 4 to 6 weeks. They offered more frequent contacts or visits for those who did not meet clinical targets or had relapses in depressive symptoms.<br>Intervention contacts and active monitoring continued for 12 months after randomization.	Collaborative care	Predominantly organisational-level intervention

Kearns et al., 2017	Policy changes to improve the current care pathway	Three potential service changes:<br>1) improving rates of opportunistic screening for depression,<br>- Opportunistic screening refers to screening for depression amongst routine primary care appointments unrelated to depression<br>- Policy to screen individuals with diabetes for depression during every primary care appointment<br>- Assumed that every primary care appointment for individuals with diabetes included an opportunistic screen for depression unless the individual had identified depression.<br>2) implementing collaborative care, and<br>- Collaborative care is an enhancement to how depression treatment is usually delivered.<br>- Requires an additional healthcare professional whose job is to improve collaboration between the individual receiving depression treatment and those delivering the depression treatment<br>- Policy of implementing collaborative care was modelled as an enhancement to the existing care pathway for individuals with depression and diabetes<br>3) Combining collaborative care with improved opportunistic screening (combination of both 1 and 2)	Collaborative care	Predominantly organisational-level intervention
Simon et al., 2007	Systematic depression treatment program	1) Three specialized nurses delivered a 12-month, stepped-care depression treatment program<br>2) Treatment program:<br>- Begin with either problem-solving treatment psychotherapy or a structured antidepressant-pharmacotherapy program<br>- Subsequent treatment (combining psychotherapy and medication, adjustments to medication, and speciality referral) was adjusted according to clinical response<br>3) Multicomponent depression management program based in the primary care clinic<br>4) Intervention was designed to serve those remaining depressed despite primary care treatment as well as those with previously unrecognized depression<br>Intervention followed a stepped-care model, with the step 1 treatment being either antidepressant pharmacotherapy or structured psychotherapy, depending on each patient's preference<br>For patients already using antidepressant medication at baseline, step 1 might include either medication adjustment or the addition of structured psychotherapy.<br>For patients not responding to step 1 treatment (i.e., Patient Health Questionnaire score failed to decrease at least 50% by 12 weeks), step 2 included addition of a second treatment modality (e.g., adding pharmacotherapy for those beginning with psychotherapy) and/or medication adjustment (e.g., dose change, medication switch, or augmentation).<br>For those not responding after an additional 12 weeks, step 3 included in-person consultation with one of the study psychiatrists and/or referral for ongoing speciality mental health care within GHC.	Collaborative care	Predominantly organisational-level intervention

Strong et al., 2008	Depression Care for People with Cancer (DCPC)	Based on an intervention for the management of depression in primary care known as collaborative care (Katon et al. 1995; Bower et al. 2006)<br>Delivered by a cancer nurse at the regional cancer centre over an average of seven sessions<br>Patients were offered a maximum of 10 one-to-one sessions over three months, preferably in person at the cancer centre but occasionally by telephone or at patients' homes if they could not attend the centre.<br><br>DCPC comprised:<br>Education about depression and its treatment (including antidepressant medication);<br>Problem-solving treatment to teach the patients coping strategies designed to overcome feelings of helplessness; and<br>Communication about the management of major depressive disorder with each patient's oncologist and primary-care doctor<br>For three months after the treatment sessions, progress was monitored by monthly telephone calls.<br>This monitoring used the nine-item Patient Health Questionnaire (PHQ-9)16 to assess the severity of depression.<br>Offered one or two additional sessions to patients who had increasing PHQ-9 scores.<br>Each 45 min treatment session was delivered by one of three cancer nurses, who followed a detailed manual.<br>All sessions were video-recorded, and 10% of sessions were randomly selected to be independently assessed for their adherence to the treatment manual<br>No further intervention was given after six months.<br><br>The nurses had no experience in psychiatry and were trained to deliver the intervention using written materials, tutorials, and supervised practice over at least three months.<br>Patients were allocated to nurses based on the nurses' workloads.<br>A psychiatrist reviewed patients' progress with the nurses every week.<br>Nurses presented each patient's scores on the Patient Health Questionnaire, their antidepressant dose, and their progress with problem-solving treatment.<br>The patient's management was then briefly discussed.<br>If the patient decided, during discussions with the nurse, to start or change antidepressant medication, they were encouraged to contact their primary care doctor for this purpose.<br>The nurse then contacted the patient's doctor before their appointment to provide information about the patient and offer advice from a study psychiatrist.	Collaborative care	Predominantly organisational-level intervention

Walker et al., 2014	Systematic integrated depression management (includes both case identification and treatment)	Combines systematic case identification by a two-stage screening system in specialist cancer clinics with a systematic collaborative care type treatment integrated with cancer care, known as Depression Care for People with Cancer (DCPC)<br>Identification process:<br>Stage 1: Screening using the Hospital Anxiety and Depression Scale (HADS) while waiting for a clinic appointment<br>Stage 2: Screening (brief diagnostic interview for major depression)<br>- Patients whose total HADS score is ≥15 are telephoned at home soon after their clinic appointment<br>- At the end of the call, patients with major depression are advised to see their primary care physician or oncology clinician, both of whom receive a report from the screening service informing them of the diagnosis of major depression.<br>Treatment process:<br>- Treatment of major depression using DCPC<br>- DCPC is a multi-component, systematic, team-delivered treatment programme integrated with the patient's cancer care.<br>- The treatment team comprises specially trained cancer nurses, consultation-liaison psychiatrists and the patient's primary care physician.<br>- The nurses provide education about depression and its treatment, deliver brief evidence-based psychological interventions (problem-solving therapy, behavioural activation) and monitor the patient's progress using the Patient Health Questionnaire nine-item (PHQ-9) depression scale.<br>- Psychiatrists supervise treatment to achieve and maintain treatment targets, advise primary care physicians about prescribing antidepressant medication and provide direct consultations to patients who are not progressing.<br>- The initial treatment phase comprises a maximum of 10 sessions with the nurse, given over four months.<br>- The patient's PHQ-9 scores are monitored monthly by telephone, and additional sessions are provided for patients who do not meet the treatment targets.	Collaborative care	Predominantly organisational-level intervention

Supplementary File 9: Details of the key design aspects of the included studies

Perspective

The viewpoint or perspective adopted to decide which types of costs and health benefits to include in an economic evaluation was reported by 16 studies.[1–16] Common perspectives adopted were societal,[1–3] payer,[4–6] and healthcare sector that includes a healthcare system,[11,12] UK NHS and Personal Social Services,[7–9] and a healthcare provider.[10]

Time horizon

Four studies had a time horizon of less than a year.[3,7,12,17] While 13 studies had a time horizon of between one and two years,[1,2,4–6,8,10,11,14–16,18,19] one had five years,[9] and other had a lifetime.[13]

Discount rate

Ten studies reported discounting.[1,2,5,7–9,12–14,17] Three UK studies used a discount rate of 3.5% for both costs and effects,[1,9,13] and one Dutch study used a discount rate of 4% for costs only.[2] Although discounting was necessary, one study[8] justified that it did not discount costs and outcomes. Five studies reported that they did not use discounting as the study duration was less than a year and thus was not applicable.[5,7,12,14,17]

Selection, measurement and valuation of outcomes

Eleven studies used QALYs only,[1,4,7–9,12,13,15–18] and one used DFDs only[6] as the outcome measure. Seven studies had two outcomes, of which five were QALYs and DFDs,[2,5,11,14,19] one used QALYs and treatment success rate,[10] and other used QALYs and treatment response rate.[3]

EQ-5D questionnaires based on patient's responses were used to capture QALYs in 10 studies.[1–3,5,7,8,14–17] One study[4] used Short-Form Health Survey (SF-12) fitted to the SF-6D utility-scale, while another used the Assessment of Quality of Life (AQoL-4D) scale.[12] Three studies obtained QALYs scores from published literature.[9,10,13] Two studies derived QALYs using DFDs.[11,19] One study converted non-preference-based scores to preference-based EQ-5D.[18] Of six studies that used DFDs as a measure of outcomes, four used the Hopkins Symptom Checklist 20 Depression Scale (HSCL-20),[6,11,14,19] one used Patient Health Questionnaire (PHQ) score,[5] and another used the Beck Depression Inventory (BDI) score.[2]

Eight studies placed the value on the health-related quality of life based on a valuation of public preferences elicited from a representative sample of the UK population,[1,3,4,7,8,14,16,17] two studies from the general Dutch population,[2,15] four from the published literature,[6,10,13,18] and one from Canadian preference scoring.[5] Four studies did not report their sources of preference data to evaluate changes in health-related quality of life.[9,11,12,19]

Costing approaches

Direct medical costs (healthcare costs related to the use of resources due to diseases or treatment) were included in all studies. These were the costs of inpatient stay, outpatient visit, emergency room visit, medications, laboratory tests, staff time (doctor, nurse, psychiatrists, psychologists, physiotherapist), or intervention (equipment and training). Seven studies considered direct non-medical costs (costs related to the treatment process), such as travel costs or informal care.[1–4,13,15,16] Four studies reported indirect costs, i.e. costs that are not directly related to treatment, such as loss of time, loss of production or pay.[2,3,13,14] In studies that considered indirect cost, productivity loss was valued using a human capital approach,[3] a friction cost method,[2] value-based pricing,[13] and due to temporary unfitness for work.[14]

Single study-based economic evaluation studies measured resources used using a variety of methods. Nine studies used self-reported questionnaires at different periods during the study,[1–3,7,8,12,14,15,18] which in some studies was supplemented by a case note review[7] or cost diary.[2,12] One study that used a questionnaire, however, measured resources used retrospectively.[15] Other studies used medical records,[4,5,11,19] administrative databases,[6,10] and service receipt inventory[16] to measure the resources. The method of measurement of resources used was unclear in one study.[17]

Eleven studies valued resources using relevant national unit costs,[1–3,7–9,12,13,15–17] which in one study was supplemented by published sources,[3] and by published sources and assumptions in two modelling studies.[9,13] Other studies used prices,[4,14] actual costs (not charges),[11,19] insurance claims data,[6,10] consensus,[5] and published sources to value resource use.[18]

The price year was explicitly reported in 16 studies and could be inferred in three studies.[6,11,19]

Consideration of uncertainty

Nine of the 14 studies based on individual patient data reported sampling uncertainty by stating confidence intervals of incremental costs and incremental effects.[5–8,11,12,15,17,19] Uncertainty around the ICER estimate was presented on a cost-effectiveness plane in seven studies,[1,3,8,12,14–16] supplemented by confidence ellipses in one study.[14] The probability of an intervention being cost-effective was presented using a cost-acceptability curve (CEAC) in 14 studies,[1–5,7,8,10–12,14–16,19] but five studies did not present CEAC.[6,9,13,17,18]

Of 16 studies that conducted a sensitivity analysis, 12 performed a one-way sensitivity analysis.[2,3,5,6,8,10–15,19] A probabilistic sensitivity analysis,[7] a multiway analysis,[17] a probabilistic sensitivity analysis along with scenario analysis,[9] and a

probabilistic sensitivity analysis along with a one-way analysis[1] was conducted by one study each. Three studies did not report any form of sensitivity analysis.[4,16,18]

Health economic analysis plan

None of the studies included in the review reported their health economic analysis plan.

Funding/Funders

Two studies did not report who funded their study,[18,19] five were funded by charity,[7,9,12,15,17] and a government or university grant funded the remaining 12 studies.[1–6,8,10,11,13,14,16]

References

1. Camacho EM, Ntais D, Coventry P, *et al.* Long-term cost-effectiveness of collaborative care (vs usual care) for people with depression and comorbid diabetes or cardiovascular disease: a Markov model informed by the COINCIDE randomised controlled trial. *BMJ Open* 2016;**6**:e012514. doi:10.1136/bmjopen-2016-012514
2. Jonkers CCM, Lamers F, Evers SMAA, *et al.* Economic evaluation of a minimal psychological intervention in chronically ill elderly patients with minor or mild to moderate depression: a randomized trial (the DELTA-study). *Int J Technol Assess Health Care* 2009;**25**:497–504. doi:10.1017/S026646230999050X
3. Nobis S, Ebert DD, Lehr D, *et al.* Web-based intervention for depressive symptoms in adults with types 1 and 2 diabetes mellitus: a health economic evaluation. *Br J Psychiatry* 2018;**212**:199–206. doi:10.1192/bjp.2018.10
4. Hay JW, Katon WJ, Ell K, *et al.* Cost-effectiveness analysis of collaborative care management of major depression among low-income, predominantly Hispanics with diabetes. *Value Health* 2012;**15**:249–54. doi:10.1016/j.jval.2011.09.008
5. Johnson JA, Lier DA, Soprovich A, *et al.* Cost-Effectiveness Evaluation of Collaborative Care for Diabetes and Depression in Primary Care. *Am J Prev Med* 2016;**51**:e13-20. doi:10.1016/j.amepre.2016.01.010
6. Simon GE, Katon WJ, Lin EHB, *et al.* Cost-effectiveness of systematic depression treatment among people with diabetes mellitus. *Arch Gen Psychiatry* 2007;**64**:65–72. doi:10.1001/archpsyc.64.1.65
7. Duarte A, Walker J, Walker S, *et al.* Cost-effectiveness of integrated collaborative care for comorbid major depression in patients with cancer. *J Psychosom Res* 2015;**79**:465–70. doi:10.1016/j.jpsychores.2015.10.012
8. Camacho EM, Davies LM, Hann M, *et al.* Long-term clinical and cost-effectiveness of collaborative care (versus usual care) for people with mental-physical multimorbidity: cluster-randomised trial. *Br J Psychiatry* 2018;**213**:456–63. doi:10.1192/bjp.2018.70
9. Walker S, Walker J, Richardson G, *et al.* Cost-effectiveness of combining systematic identification and treatment of co-morbid major depression for people with chronic diseases: the example of cancer. *Psychol Med* 2014;**44**:1451–60. doi:10.1017/S0033291713002079
10. Pan Y-J, Kuo K-H, Chan H-Y, *et al.* Cost-effectiveness and cost-utility of selective serotonin reuptake inhibitors, serotonin norepinephrine reuptake inhibitors, and tricyclic antidepressants in depression with comorbid cardiovascular disease. *J Psychiatr Res* 2014;**54**:70–8. doi:10.1016/j.jpsychires.2014.03.002
11. Katon W, Russo J, Lin EHB, *et al.* Cost-effectiveness of a multicondition collaborative care intervention: a randomized controlled trial. *Arch Gen Psychiatry* 2012;**69**:506–14. doi:10.1001/archgenpsychiatry.2011.1548
12. Moayeri F, Dunt D, Hsueh Y-SA, *et al.* Cost-utility analysis of telephone-based cognitive behavior therapy in chronic obstructive pulmonary disease (COPD) patients with anxiety and depression comorbidities: an application for willingness to accept concept. *Expert Rev Pharmacoecon Outcomes Res* 2019;**19**:331–40. doi:10.1080/14737167.2019.1536550
13. Kearns B, Rafia R, Leaviss J, *et al.* The cost-effectiveness of changes to the care pathway used to identify depression and provide treatment amongst people with diabetes in England: a model-based economic evaluation. *BMC Health Serv Res* 2017;**17**:78. doi:10.1186/s12913-017-2003-z
14. Aragonès E, Sánchez-Iriso E, López-Cortacans G, *et al.* Cost-effectiveness of a collaborative care program for managing major depression and chronic musculoskeletal pain in primary care: Economic evaluation alongside a randomized controlled trial. *J Psychosom Res* 2020;**135**:110167. doi:10.1016/j.jpsychores.2020.110167
15. Goorden M, van der Feltz-Cornelis CM, van Steenbergen-Weijenburg KM, *et al.* Cost-utility of collaborative care for the treatment of comorbid major depressive disorder in outpatients with chronic physical conditions. A randomized controlled trial in the general hospital setting (CC-DIM). *Neuropsychiatr Dis Treat* 2017;**13**:1881–93. doi:10.2147/NDT.S134008
16. Barley EA, Walters P, Haddad M, *et al.* The UPBEAT nurse-delivered personalized care intervention for people with coronary heart disease who report current chest pain and depression: a randomised controlled pilot study. *PLoS One* 2014;**9**:e98704. doi:10.1371/journal.pone.0098704
17. Strong V, Waters R, Hibberd C, *et al.* Management of depression for people with cancer (SMaRT oncology 1): a randomised trial. *Lancet* 2008;**372**:40–8. doi:10.1016/S0140-6736(08)60991-5
18. Basu R, Ory MG, Towne SD, *et al.* Cost-effectiveness of the chronic disease self-management program: implications for community-based organizations. *Front Public Health* 2015;**3**:27. doi:10.3389/fpubh.2015.00027
19. Katon W, Unützer J, Fan M-Y, *et al.* Cost-effectiveness and net benefit of enhanced treatment of depression for older adults with diabetes and depression. *Diabetes Care* 2006;**29**:265–70. doi:10.2337/diacare.29.02.06.dc05-1572

Supplementary File 10: Economic evidence profile

Study	Limitations	Other comments	Incremental			Uncertainty
			Costs*	Effects	Cost-effectiveness ratio (ICER)*	
**Level of healthcare provision: Patient-level intervention**						
Type of intervention: Self-management support intervention						
Basu et al., 2015<br><br>Location and setting: USA, 17 States (22 organizations)	Very serious limitations[c]	Study employed a pre-post longitudinal design with a 1-year time horizon. No comparison group. Excludes potential costs.<br><br>Intervention: Chronic Disease Self-Management Program (CDSMP)<br><br>Comparator: No intervention<br><br>Price year: 2010	£189 (US$219) (min cost)	0.006 QALYs	£31,540 (US$36,500) per QALY gained	ICER by baseline depression status indicates that it will cost more per QALYs gained for those diagnosed with depression (from $36,500 to $97,166) based on their Patient Health Questionnaire-8 score.
Jonkers et al., 2009<br><br>Location and setting: Netherlands, South of the Netherlands (89 primary care practices)	Minor limitations[a]	Study employed a two-armed randomised controlled trial with a 1-year time horizon<br><br>Intervention: Minimal Psychological Intervention (MPI)<br><br>Comparator: Usual care<br><br>Price year: 2004	Not reported	Not reported	<u>Based on DFDs:</u><br>a) -£16 (-€14) per DFDs<br><br><u>Based on QALYs:</u><br>a) -£12,962 (-€11,508; 95% CI: −160,502 to 192,027) per QALY-EQ5D (Dominant)<br><br>b) -£14,118 (-€12,534; 95% CI: −190,366 to 101,049) per QALY-DFD (Dominant)	82% probability of the MPI being cost-effective at €20,000 per QALY gained<br><br>89% probability of the MPI being cost-effective is at €80,000 per QALY gained<br><br>Complete case analysis showed an increase of the probability of the MPI being less effective and less costly (30%), while the probability of the MPI being costlier but also more effective decreased (3%).
Nobis et al., 2018<br><br>Location and setting: Germany	Potentially serious limitations[b]	Study employed a randomised control trial with a 6-month time horizon. Not possible to draw conclusions about the longer-term follow-up<br><br>Intervention: Web-based intervention i.e., GET.ON	£102 (€97)	<u>Based on treatment response:</u><br>0.42 treatment response<br><br><u>Based on QALYs:</u><br>0.01 QALYs	<u>Based on treatment response:</u><br>£245 (€233) per treatment response<br><br><u>Based on QALYs:</u><br>£11,274 (€10,708) per QALY gained	<u>For treatment response:</u><br>54% probability that the intervention generates better clinical outcomes, but the intervention is also associated with additional costs.<br><br>46% probability that better health

		Mood Enhancer Diabetes (GET.ON M.E.D.)<br><br>Comparator: Web-based psychoeducation<br><br>Price year: 2013				outcomes are achieved for lower costs in the intervention group.<br><br>97% probability of the intervention being cost-effective at €5,000 for a treatment response<br><br>For QALYs:<br>37% probability that the intervention generates more QALYs – but at higher costs – compared with the control<br><br>13% probability that the intervention is both less costly and more effective.<br><br>46% probability that the intervention should be regarded as more cost-effective at 0 WTP<br><br>51% probability that the intervention is cost-effective at €14,000 for an additional QALY,
Type of intervention: Telephone-based cognitive behavioural therapy						
Moayeri et al., 2018<br><br>Location and setting: Australia, Melbourne (four tertiary hospitals and pulmonary rehabilitation programs)	Very serious limitations[c]	Study employed a pragmatic, two-armed randomised control trial with a 17-week (relatively short) time horizon<br><br>Intervention: Telephone-based cognitive behavioural therapy (TB-CBT)<br><br>Comparator: Standard care plus placebo-befriending phone calls<br><br>Price year: 2013	£-226 (AUS$-407.3; 95% CI: -338.6 to -475.0)	−0.0081 (95% CI: -0.0081 to 0.1065) QALYs	£27,958 (AUS$50,284;95% CI: 13,426 to −32,018) per QALY gained	If the societal' s minimum (flooring threshold) willingness-to-accept (WTA) is AUS$64,000 per QALY forgone, the probability of TB-CBT being cost-effective was 42%<br><br>With a probability of 83%, TB-CBT would be less costly but also have lower utility and with 17% chance it would be dominant.<br><br>The result of this study was not sensitive to the change of assumptions tested in this sensitivity analysis.
Type of intervention: Antidepressants treatment						

Pan et al., 2014<br><br>Location and setting: Taiwan	Very serious limitations[c]	Study employed an observational (administrative database) study with a 18-month time horizon.<br><br>Intervention: Three antidepressants:<br>1) Selective serotonin reuptake inhibitors (SSRIs)<br>2) Serotonin norepinephrine reuptake inhibitors (SNRIs)<br>3) Tricyclic antidepressants (TCAs)<br><br>Comparator: SSRIs, SNRIs, TCAs<br><br>Price year: 2003/2004	a) Selective Serotonin Reuptake Inhibitors (SSRIs) compared to Serotonin Norepinephrine Reuptake Inhibitors (SNRIs): £-426 (NTD -8,376)<br><br>b) SSRIs compared to Tricyclic Antidepressants (TCAs): £166 (NTD 3,269)<br><br>c) SNRIs compared to TCAs: £592 (NTD 11,645)	Based on treatment success rate (for patients with cardiovascular disease):<br>a) SSRIs compared to SNRIs: 0.01 percentage point of treatment success<br><br>b) SSRIs compared to TCAs: 0.03 percentage point of treatment success<br><br>c) SNRIs compared to TCAs: 0.02 percentage point of treatment success<br><br>Based on QALYs (for patients with cardiovascular disease):<br>a) SSRIs compared to SNRIs: 0.002 QALYs<br><br>b) SSRIs compared to TCAs: 0.003 QALYs<br><br>c) SNRIs compared to TCAs: 0.001 QALYs	Based on treatment success rate (for patients with cardiovascular disease):<br>a) SSRIs compared to SNRIs: Dominant<br><br>b) SSRIs compared to TCAs: £55 (NTD 1,083) per percentage point of treatment success<br><br>c) SNRIs compared to TCAs: £296 (NTD 5,823) per percentage point of treatment success<br><br>Based on QALYs (for patients with cardiovascular disease):<br>a) SSRIs compared to SNRIs: Dominant<br><br>b) SSRIs compared to TCAs: £55,394 (NTD 1.09 million) per QALY gained<br><br>c) SNRIs compared to TCAs: £592,028 (NTD 11.6 million) per QALY gained	For those with CVD, if society is willing to pay NTD 1.5 million for an additional QALY, there is a 68.9% (psychiatric costs) and 46.1% (total costs) likelihood that SSRIs would be the most cost-effective compared to TCAs and SNRIs.<br><br>For those with CVD, if society is willing to pay NTD 2.0 million for an additional QALY, there is a 91.7% (psychiatric costs) and 68.8% (total costs) likelihood that SSRIs would be the most cost-effective compared to TCAs and SNRIs.
**Level of healthcare provision: Organisational-level intervention**						
Type of intervention: Collaborative care (for people with depressive disorder and diabetes)						

Katon et al., 2006<br><br>Location and setting: USA, 18 primary care clinics from eight health care organizations in five states	Potentially serious limitations[b]	Study employed a randomised controlled trial with a 2-year time horizon. Estimate of QALY is not from validated measure.<br><br>Intervention: Improving Mood-Promoting Access to Collaborative Trial (IMPACT)<br><br>Comparator: Usual care<br><br>Type of intervention: Collaborative care<br><br>Price year: Not reported (implied 2001)	£26 (US$25; 95% CI: -1,638 to 1,689)	Based on DFDs:<br>a) 115.4 (95% CI: 71.7 to 159.1) DFDs<br><br>Based on QALYs:<br>a) 0.126 (95% CI: 0.079 to 0.174) QALYs<br><br>b) 0.063 (95% CI: 0.039 to 0.087) QALYs	Based on DFDs:<br>< £1 (25 cents; 95% CI: -$14 to $15) per DFDs<br><br>Based on QALYs:<br>a) £206 (US$198; 95% CI: 144 to 316) per QALY gained<br><br>b) £413 (US$397; 95% CI: 287 to 641) per QALY gained<br><br>Incremental net benefit:<br>£1,175 (US$1,129; 95% CI: 692 to 1,572)	Based on total outpatient costs, the probability that the intervention improved outcomes and saved money was estimated by bootstrapping procedures to be 50.3%.<br><br>When total costs (inpatient and outpatient) are included, the probability that the intervention improved outcomes and saved money was 67.3%.<br><br>At Willingness to pay of US$5 per day incremental net benefit is US$552 (95% CI: 334 to 771).
Simon et al., 2007<br><br>Location and setting: USA, Western Washington (9 primary care clinics)	Minor limitations[a]	Study employed a randomised controlled trial with a 2-year time horizon among outpatients<br><br>Intervention: Systematic depression treatment program<br><br>Comparator: Usual care<br><br>Type of intervention: Collaborative care<br><br>Price year: Not reported (implied 2001/2002)	£-327 (US$-314; 95% CI: -1007 to 379)	£-327 (US$-314; 95% CI: -1007 to 379)	£-5.4 (US$ -5.2; 95% CI: -17.6 to 7.2) per DFDs (Dominant)	including only participants with complete follow-up data (i.e., completed the 24-month assessment and remained in the health plan for 24 months) yielded identical results for incremental effectiveness; adjusted cost savings was somewhat greater (US$ -605 (95% CI, -$1766 to $566)<br><br>If we attach no value (i.e., willingness to pay=US$0) to a day free of depression, then the incremental net benefit of the intervention program is equal to cost savings alone, approximately US$300 per patient treated.<br><br>Incremental net benefit increases as we attach greater benefit to a day free of depression: approximately US$630 per patient if we value an additional day free of depression at US$5, approximately US$950 for a value of US$10, and approximately US$1600 for a value of US$20.

						The 95% CI for incremental net benefit excludes zero for any value of willingness to pay greater than US$8 per additional depression-free day<br><br>Among those not using antidepressants prior to enrolment, the gain in depression-free days was 84 (95% CI, 52 to 116) and estimated cost savings were US$421 (95% CI, $1324 decrease to US$483 increase in cost).<br><br>Among those already receiving depression treatment, the intervention group experienced 34 (95% CI, 5 to 63) additional days free of depression and a US$30 increase in outpatient costs (95% CI, US$970 decrease to US$1030 increase).
Hay et al., 2012<br><br>Location and setting: USA, Los Angeles County public community clinics	Potentially serious limitations[b]	Study employed a randomised controlled trial with 18 months time horizon. Statistically significant imbalance between study groups at baseline randomisation<br><br>Intervention: Multifaceted Diabetes and Depression Program (MDDP)<br><br>Comparator: Enhanced usual care<br><br>Type of intervention: Collaborative care<br><br>Price year: 2009	£450 (US$515)	0.13 QALYs	£3,543 (US$4,053) per QALY gained	More than a 50% probability that the MDDP was cost-effective at a threshold willingness-to-pay of US$5,000 per QALY and more than a 90% probability that the MDDP intervention was cost-effective at a willingness-to-pay threshold of US$12,000 per QALY

Johnson et al., 2016<br><br>Location and setting: Canada, Alberta (in four primary care networks)	Potentially serious limitations[b]	Study employed a controlled implementation trial with a 1-year time horizon. Comparison groups were not randomly allocated, but rather the study used a monthly time series (on-off design)<br><br>Intervention: Collaborative care<br><br>Comparator: Enhanced care and Usual care<br><br>Price year: 2011	a) Collaborative care compared with enhanced care: £389 (C$571; 95% CI: -3,129 to 4,241)<br><br>b) Collaborative care compared with usual care: £695 (C$1,021; 95% CI: -2,750 to 4,775)<br><br>c) Enhanced care compared with usual care: £307 (C$450; 95% CI: -3,814 to 4,727)	Based on DFDs:<br>a) Collaborative care compared with enhanced care: 51.7 (95% CI: 15.9 to 87.3) DFDs<br><br>b) Collaborative care compared with usual care: 117.6 (95% CI: 87.0 to 148.1) DFDs<br><br>c) Enhanced care compared with usual care: 65.9 (95% CI: 31.8 to 100.2) DFDs<br><br>Based on QALYs:<br>a) Collaborative care compared with enhanced care: 0.036 (95% CI: -0.023 to 0.095) QALYs<br><br>b) Collaborative care compared with usual care: 0.042 (95% CI: -0.011 to 0.096) QALYs<br><br>c) Enhanced care compared with usual care: 0.006 (95% CI: -0.067 to 0.069) QALYs	Based on DFDs:<br>a) Collaborative care compared with enhanced care:<br>£7 (C$11) per DFDs<br><br>b) Collaborative care compared with usual care: £6 (C$9) per DFDs<br><br>c) Enhanced care compared with usual care: £5 (C$7) per DFDs<br><br>Based on QALYs:<br>a) Collaborative care compared with enhanced care:<br>£10,803 (C$15,861) per QALY gained<br><br>b) Collaborative care compared with usual care: £16,597 (C$24,368) per QALY gained<br><br>c) Enhanced care compared with usual care: £51,949 (C$76,271) per QALY gained	The cost-effectiveness acceptability curve (Figure 1A) indicates that the likelihood of the collaborative care intervention being cost-effective is higher than both alternatives at willingness-to-pay levels lower than a threshold of C$40 per DFD. As society's willingness to pay for an additional DFD increases beyond a threshold of C$40 per DFD, the probability that collaborative care is cost-effective increases steadily.<br><br>Furthermore, the acceptability curve for collaborative care (Figure 1B) indicates a greater likelihood of being cost-effective at commonly considered thresholds, and increases steadily as the level of society's willingness to pay increases.

Kearns et al., 2017<br><br>Location and setting: UK, England (primary care)	Minor limitations[a]	Study employed a mathematical model using discrete event simulation with a lifetime horizon<br><br>Intervention: Three policy changes to improve the current care pathway (implementing collaborative care, improving opportunistic screening, and combining collaborative care with improved opportunistic screening)<br><br>Comparator: Improved opportunistic screening, current practice, combined policy (combining collaborative care with improved opportunistic screening)<br><br>Price year: 2013	a) Collaborative care compared with improved opportunistic screening: £-4.45 billion (£-3.80 billion)<br><br>b) Collaborative care compared to current practice: £1.23 billion (£1.05 billion)<br><br>c) Combined policy compared to collaborative care alone: £6.75 billion (£5.76 billion)	a) Collaborative care compared with improved opportunistic screening: 21000 QALYs<br><br>b) Collaborative care compared to current practice: 97000 QALYs<br><br>c) Combined policy compared to collaborative care alone: 85000 QALYs	a) Collaborative care dominated improved opportunistic screening<br><br>b) Collaborative care compared to current practice: £12,656 (£10,798) per QALY gained<br><br>c) Combined policy compared to collaborative care alone: £79,723 (£68,017) per QALY gained	the cost-effectiveness results were most sensitive to the estimated time until relapse, and the hazard ratio for depression affecting diabetes-related complications<br><br>If only depression outcomes had been considered, then the ICERs compared with usual practice (£17,000, £91,000 and £50,000 for policies 1 (Collaborative care), 2 (Opportunistic screening) and 3 (both collaborative care and opportunistic screening) respectively would have been higher than when considering both diabetes and depression. Hence, if only outcomes relating to depression were considered then the cost-effectiveness of each of the policies would have been under-estimated.
Type of intervention: Collaborative care (for people with comorbid major depression and cancer)						
Strong et al., 2008<br><br>Location and setting: UK, Scotland (regional tertiary NHS cancer centre)	Potentially serious limitations[b]	Study employed a randomised controlled trial with a 6-month time horizon<br><br>Intervention: Depression Care for People with Cancer (DCPC)<br><br>Comparator: Usual care<br><br>Price year: 2006	£450 (£334.86; 95% CI: £276 to £393)	0.063 (95% CI: 0.032 to 0.095) QALY	£7,098 (£5,278) per QALY gained	A conservative sensitivity analysis, taking the lower limit of the 95% CI for the effect size (0.032 QALYs) and the upper limit for the additional cost (£393) gives a cost of £12,300 per QALY gained.<br><br>Taking the upper limit for the effect size (0.095 QALYs) and the lower limit for the additional cost (£276) gives a cost of £2,900 per QALY gained.
Duarte et al., 2015<br><br>Location and setting: UK, Scotland (3 cancer centres and their associated clinics (Glasgow, Edinburgh and Dundee))	Potentially serious limitations[b]	Study employed a multicentre randomised controlled trial with a 48 weeks time horizon<br><br>Intervention: DCPC<br><br>Comparator: Usual care	£780 (£631; 95% CI: 595.37 to 667.24)	0.066 (95% CI: 0.031 to 0.101) QALYs	£11,802 (£9,549) per QALY gained	The probability of DCPC being cost-effective was 0.9 or greater at cost-effectiveness thresholds above £20,000 per QALY for the base case and scenario analyses.

		Price year: 2010/2011				
Walker et al., 2014<br><br>Location and setting: UK	Minor limitations[a]	Study employed a decision analytic model with a 5-year time horizon<br><br>Intervention: Systematic integrated depression management (that includes DCPC)<br><br>Comparator: Usual practice<br><br>Price year: 2010	£122 (£98.34)	0.0084 QALYs	£14,540 (£11,765) per QALY gained	>99% probability that systematic depression management is cost-effective at £20,000 per QALY<br><br>The results were consistent across sex and age.<br><br>Varying the estimated incidence of major depression had little effect on cost-effectiveness; doubling the incidence to 4.2% only slightly reduced the ICER to £11,278 per QALY gained<br><br>The probability of systematic management being cost-effective remained more than 99%, regardless of the time horizon considered.<br><br>Even if the estimated sensitivity and specificity of usual identification were increased to an improbable 100%, usual practice was still not cost-effective at commonly accepted thresholds.<br><br>Using the estimate of treatment effectiveness from other trials of collaborative care treatment of depression in primary care did not significantly change the results and generated an ICER of £10,546 per QALY.
Type of intervention: Collaborative care (for people with depression and multiple long-term conditions)						

Katon et al., 2012<br><br>Location and setting: USA, Washington (fourteen primary care clinics of an integrated health care system)	Potentially serious limitations[b]	Study employed a randomised controlled trial with a 2-year time horizon. Estimate of QALY is not from validated measure rather based on clinical outcomes. Substantial uncertainty around both costs and outcomes<br><br>Intervention: TEAMcare<br><br>Comparator: Usual primary care<br><br>Price year: Not reported (implied 2009)	£-519 (-US$594; 95% CI: -$3421 to $2053)	Based on DFDs:<br>114 (95% CI: 79 to 149) DFDs<br><br>Based on QALYs:<br>0.335 (95% CI: -0.18 to 0.85) QALYs	Based on DFDs:<br>-£5 (-US$5.26; 95% CI: -$29.76 to $19.17) per DFDs (Dominant)<br><br>Based on QALYs:<br>-£1,550 (-US$ 1,773; 95% CI: -$2878 to $2878) per QALY gained (Dominant)	The sensitivity analysis that allowed for reimbursement for diabetes nurse visits at US$54 per visit for up to 10 visits showed even more favourable incremental 24-month total outpatient cost savings of US$1116 (95% CI, −$3768 to $1536), as well as cost savings of US$9.88 (95% CI, −$34.97 to $14.16) per DFDs and $3297 (95% CI, −$4014 to $2722) per QALY gained.<br><br>The cost-effectiveness acceptability analysis found that there was a 99.7% probability that the total 24-month outpatient costs would be less than US$20,000 per QALY.
Goorden et al., 2017<br><br>Location and setting: Netherlands (5 general hospitals in Amsterdam, Almelo, Hengelo, Ede, and Maastricht)	Potentially serious limitations[b]	Study employed a multicentre randomised controlled trial with a 1-year time horizon. Small sample size (81 patients)<br><br>Intervention: Collaborative care<br><br>Comparator: Usual care<br><br>Price year: 2016	Healthcare perspective:<br>£1,892 (€1,939; 95% CI: -1,751 to 6,428)<br><br>Societal perspective:<br>£1,639 (€1,680; 95% CI: -1,951 to 5,911)	0.07 (95% CI: -0.002 to 0.14) QALYs	Healthcare perspective:<br>£27,674 (€28,366) per QALY gained<br><br>Societal perspective:<br>£24,088 (€24,690) per QALY gained	Healthcare perspective:<br>At a threshold of €20,000/QALY, there is 40% probability that the intervention is accepted. At an ICER of €60,000/QALY, there is ~80% probability that the intervention is accepted.<br><br>Societal perspective:<br>At a threshold of €20,000/QALY, there is ~60% probability that the intervention is accepted. At an ICER of €60,000, there is ~80% probability that the intervention is accepted.

Camacho et al., 2016<br><br>Location and setting: UK, North West of England (36 primary care (general) practices)	Potentially serious limitations[b]	Study employed a Markov decision-analytic model informed by the randomised controlled trial with a 2-year time horizon. Parameters used in the model were derived from a single within-trial data. Extrapolation of short-term (4-month) trial data to estimate cost-effectiveness over 24 months.<br><br>Intervention: Collaborative care<br><br>Comparator: Usual care<br><br>Price year: 2014/2015	£777 (£674)	0.04 QALYs	£18,580 (£16,123) per QALY gained	The probability that collaborative care is cost-effective (vs usual care) was 0.53 at a willingness to pay threshold (WTPT) of £20,000 and 0.60 at a WTPT of £60,000. The probability that collaborative care was cost-effective fell below 0.5 at a WTPT of £7,000.
Camacho et al., 2018<br><br>Location and setting: UK, North West of England (36 primary care (general) practices)	Minor limitations[a]	Study employed a cluster randomised trail with a 2-year time horizon<br><br>Intervention: Collaborative care<br><br>Comparator: Usual care<br><br>Price year: 2015/2016	£2039 (£1,777; 95% CI: −320 to 3,875)	0.136 (95% CI: 0.061–0.212) QALYs	£14,995 (£13,069) per QALY gained	75% probability of being cost-effective at £20,000 and at £30,000 the probability that collaborative care is more cost-effective than usual care is 92%
Type of intervention: Collaborative care (for people with major depression and chronic musculoskeletal pain)						
Aragonès et al., 2020<br><br>Location and setting: Spain, Catalonia (eight urban primary care centres)	Minor limitations[a]	Study employed a randomised controlled trial with a 1-year time horizon<br><br>Intervention: DepRessiOn and Pain (DROP)<br><br>Comparator: Usual care<br><br>Price year: 2016	<u>Healthcare system perspective:</u> £278 (€234)<br><br><u>Societal perspective:</u> £279 (€235)	<u>Based on DFDs:</u> 8.12 DFDs<br><br><u>Based on QALYs:</u> 0.009 QALYs	<u>Based on DFDs:</u> Healthcare system perspective: £34 (€29) per DFDs<br><br>Societal perspective: £34 (€29) per DFDs<br><br><u>Based on QALYs:</u> Healthcare system perspective: £28,495 (€23,989) per QALY gained<br><br>Societal perspective:	50% probability of the program being cost-effective at €23,989/QALY (healthcare system perspective)<br><br>Although the willingness to pay increased indefinitely, the increase in the probability of cost-effectiveness was slight and remained under 60%, illustrating the high degree of uncertainty surrounding the results.<br><br>In the DFD analysis, the 50% probability of the intervention

					£28,629 (€24,102) per QALY gained	being cost-effective was achieved at a willingness to pay of €29/DFD, but this probability did not exceed 70% at any of the levels of greater willingness<br><br>In the sensitivity analysis (i.e., from the complete cases), intervention is dominant for both the ICERs (QALYS and DFDs) from the societal perspective.
Type of intervention: Self-management (for people with major depression and coronary heart disease)						
Barley et al., 2014<br><br>Location and setting: UK, South London (17 general practices)	Minor limitations[a]	Study employed a randomised controlled trial (pilot study) with a 1-year time horizon<br><br>Intervention: Personalized care i.e., UPBEAT<br><br>Comparator: Treatment as usual<br><br>Price year: 2010	Not reported	0.038 QALYs	£36,979 (£29,921) per QALY gained	Personalised care appeared to be more cost-effective up to a QALY threshold of £3,035

Note:
*Costs reported in original study were converted to 2022 UK pounds (£). All costs were converted to 2022 UK Pounds by applying the GDP deflator index and purchasing power parities conversion rate to compare the costs and incremental cost-effectiveness analysis (expressed in different currencies and/ or price years in the included studies) using the Campbell and Cochrane Economics Methods Group (CCEMG) – Evidence for Policy and Practice Information and Coordinating Centre (EPPI-Centre) Cost Converter (v.1.6.)
The ICER value shown may be different because of round-ups in costs and effects.
NTD, New Taiwan Dollar; QALY, Quality-adjusted life years; ICERs, Incremental Cost-effectiveness Ratio; DFDs, Depression-free days
[a]Minor limitations – the study meets all quality criteria or fails to meet 1 or more quality criteria, but this is unlikely to change the conclusions about cost-effectiveness;
[b]Potentially serious limitations – the study fails to meet 1 or more quality criteria, and this could change the conclusions about cost-effectiveness;
[c]Very serious limitations – the study fails to meet 1 or more quality criteria, and this is highly likely to change the conclusions about cost-effectiveness. Such studies would usually be excluded from the review.
